# Stable glioma incidence and increased patient survival over the past two decades in Norway: a nationwide registry-based cohort study

**DOI:** 10.2340/1651-226X.2024.24970

**Published:** 2024-03-19

**Authors:** Erlend Skaga, Cassia B. Trewin-Nybråten, Pitt Niehusmann, Tom Børge Johannesen, Kirsten Marienhagen, Leif Oltedal, Stephanie Schipmann, Anne Jarstein Skjulsvik, Ole Solheim, Tora Skeidsvoll Solheim, Terje Sundstrøm, Einar O. Vik-Mo, Petter Brandal, Tor Ingebrigtsen

**Affiliations:** a Vilhelm Magnus Laboratory for Neurosurgical Research and Department of Neurosurgery, Oslo University Hospital, Oslo, Norway; b Department of Pathology, Beth Israel Deaconess Medical Center and Harvard Medical School, Boston, USA; c Department of Registration, Cancer Registry of Norway, Norwegian Institute of Public Health, Oslo, Norway; d Department of Pathology, Oslo University Hospital, Oslo, Norway; e Department of Oncology, University Hospital of North Norway, Tromsø, Norway; f Mohn Medical Imaging and Visualization Centre, Department of Radiology, Haukeland University Hospital, Bergen, Norway; g Department of Clinical Medicine, University of Bergen, Bergen, Norway; h Department of Neurosurgery, Haukeland University Hospital, Bergen, Norway; i Medical Faculty, University Hospital Muenster, Germany; j Department of Pathology, St. Olavs Hospital, Trondheim University Hospital, Trondheim, Norway; k Department of Clinical and Molecular Medicine, Faculty of Medicine and Health Sciences, Norwegian University of Science and Technology, Trondheim, Norway; l Department of Neurosurgery, St. Olavs University Hospital, Trondheim, Norway; m Department of Neuromedicine and Movement Science, Norwegian University of Science and Technology, Trondheim, Norway; n Cancer Clinic, St. Olavs University Hospital, Trondheim, Norway; o Department of Clinical and Molecular Medicine, Faculty of Medicine and Health Sciences, Norwegian University of Science and Technology, Trondheim, Norway; p Department of Clinical Medicine, University of Bergen, Bergen, Norway; q Institute for Clinical Medicine, Faculty of Medicine, University of Oslo, Oslo, Norway; r Department of Oncology, Division of Cancer Medicine, Oslo University Hospital, Oslo, Norway; s Institute for Cancer Genetics and Informatics, Oslo University Hospital, Oslo, Norway; t Department of Clinical Medicine, Faculty of Health Sciences, UiT the Arctic University of Norway, Tromsø, Norway; u Department of Neurosurgery, Otorhinolaryngology and Ophthalmology, University Hospital of North Norway, Tromsø, Norway

**Keywords:** Cancer registries, glioma, cancer incidence, cancer survival, glioma epidemiology

## Abstract

**Background:**

Surveillance of incidence and survival of central nervous system tumors is essential to monitor disease burden and epidemiological changes, and to allocate health care resources. Here, we describe glioma incidence and survival trends by histopathology group, age, and sex in the Norwegian population.

**Material and methods:**

We included patients with a histologically verified glioma reported to the Cancer Registry of Norway from 2002 to 2021 (*N* = 7,048). Population size and expected mortality were obtained from Statistics Norway. Cases were followed from diagnosis until death, emigration, or 31 December 2022, whichever came first. We calculated age-standardized incidence rates (ASIR) per 100,000 person-years and age-standardized relative survival (RS).

**Results:**

The ASIR for histologically verified gliomas was 7.4 (95% CI: 7.3–7.6) and was higher for males (8.8; 95% CI: 8.5–9.1) than females (6.1; 95% CI: 5.9–6.4). Overall incidence was stable over time. Glioblastoma was the most frequent tumor entity (ASIR = 4.2; 95% CI: 4.1–4.4). Overall, glioma patients had a 1-year RS of 63.6% (95% CI: 62.5–64.8%), and a 5-year RS of 32.8% (95% CI: 31.6–33.9%). Females had slightly better survival than males. For most entities, 1- and 5-year RS improved over time (5-year RS for all gliomas 29.0% (2006) and 33.1% (2021), *p* < 0.001). Across all tumor types, the RS declined with increasing age at diagnosis.

**Interpretation:**

The incidence of gliomas has been stable while patient survival has increased over the past 20 years in Norway. As gliomas represent a heterogeneous group of primary CNS tumors, regular reporting from cancer registries at the histopathology group level is important to monitor disease burden and allocate health care resources in a population.

## Introduction

Gliomas are a group of primary central nervous system (CNS) neoplasms with glial characteristics primarily arising in the brain. Although gliomas are the most common malignant brain tumor in adults, they are rare, and account for less than 2% of all neoplasms typically included in cancer registries [1, 2]. In the latest WHO classification, more than 40 subtypes of gliomas have been defined; subtypes that span a range of biological aggressiveness with highly variable incidence and post diagnostic life expectancy [3].

The Nordic countries have previously reported higher incidence rates than other western regions [4–8]. Nordic populations may have higher underlying risk due to patient characteristics such as predominantly non-Hispanic white ethnicity [9], or higher detection rates due to healthcare system characteristics such as tax-based funding with broad universal access to neuroimaging and treatment [5]. Life expectancies vary widely by tumor type and established prognostic factors such as age and intensity of treatment [10, 11]. In a population, a precise description of patterns and trends of incidence and survival is essential to estimate the disease burden, allocate resources and plan healthcare services, control programs and research activities.

The Cancer Registry of Norway (CRN) monitors the total cancer burden in the population. One objective of the CRN is to summarize current descriptive epidemiology, including that of primary brain and other CNS neoplasms [2]. Norway has a well-defined population of around 5.5 million people with unique national ID-numbers and a universal tax-funded health care system, well suited for providing precise descriptive epidemiology of cancer from an entire population. Here, we report the most recent data on patterns and trends of incidence and survival of histologically verified gliomas from the entire Norwegian population spanning the past 20 years.

## Material and methods

### Study design

The study was a population-wide registry-based cohort study of all patients reported to the CRN and diagnosed with a histologically verified gliomas from the entire Norwegian population from 2002 to 2021. The study was reported in accordance with the STROBE guidelines.

### Data sources

We used data from the CRN to identify cases. Since 1953, reporting of all new cancer cases has been mandatory by law. The registry is based on pathology reports sent automatically from pathology departments, clinical reports sent manually by hospitals, autopsy reports and death certificates. The workflow of data into CRN is illustrated in Supplementary Figure 1. Data from the CRN undergo strict quality controls, and the CRN is recognized to contain data of high quality and completeness, estimated at > 98.5% for all cancer sites (C00–C96) during 2001–2005 [12] and 2018–2022 [2]. The CRN data are thus valid for population studies [12]. The CRN estimates completeness by the capture-recapture method [13, 14], where the number of cases registered by two data sources, namely (1) pathology reports and/or death certificates, and (2) clinical notifications, was used to estimate how many cases were missing from the CRN, assuming the two data sources were reported independently. For cases included in this study, we estimated completeness to be 98.8% (Supplementary Table 1). The CRN also receives regular data updates from Statistics Norway’s Central Population Registry, with information about deceased persons, annual population counts (used for calculating incidence rates) and mortality rates (used for estimating relative survival [RS]), stratified by age, sex, calendar year and residential health trust. The data were updated to 31 December 2022 in this study.

### Study population

The target population was all Norwegian inhabitants with a histologically verified primary glioma diagnosed during 2002–2021. Our target population was chosen on the basis that histologically verified cases were practically complete in the CRN throughout the study period, whereas clinical reporting of cases with no histological specimen was poorer and variable over time. We identified 7,713 cases reported to CRN and excluded 628 (8.1%) that were not histologically verified (only clinically reported), and 37 (0.5%) that were reported from autopsy or death certificate only. Our final study population included 7,048 cases.

### Classification by histopathological group and behavior

As there is no standard definition, we defined gliomas as International Classification of Diseases in Oncology 3rd Edition (ICD-O-3) topography codes C71–C72 and histopathology codes 9380–9385, 9391–9460, 9505 and 9509 (Supplementary Table 2). Gliomas were then grouped according to the Central Brain Tumor Registry of the United States (CBTRUS) tumor histopathology groupings [15] as follows: glioblastoma, anaplastic astrocytoma, diffuse astrocytoma, oligoastrocytic tumor, anaplastic oligodendroglioma, oligodendroglioma, pilocytic astrocytoma, unique astrocytoma variant, ependymal tumor, or neuronal and mixed neuronal-glial tumor. In line with CBTRUS, we also included a miscellaneous category (other glioma). Although molecular markers such as isocitrate dehydrogenase 1/2 mutations or 1p19q co-deletions were incorporated into the 2016 WHO classification, these biomarkers were not incorporated into the CRN until recently, so tumors could not be subgrouped by these markers in the present study.

Gliomas can be broadly classified as non-malignant (ICD-O-3 behavior code of /0 for benign and /1 for uncertain), and malignant (ICD-O-3 behavior code /3). In contrast to CBTRUS, pilocytic astrocytoma was reported as a non-malignant tumor entity in the CRN. The number of cases per morphology code and year are reported in Supplementary Table 2 and 3, respectively.

### Statistical methods

Statistical analyses and graphic presentation were done with STATA v17.0 and Keynote v12.1. Patient and tumor characteristics presented in this study were complete for all cases. For each histopathology group, we presented number and percentage of patients by ICD-O-3 behavior code, sex, and age group, and estimated median age at diagnosis. We also estimated incidence rates, RS, median survival, and overall survival for histopathology groups, stratified by sex, age and diagnosis period, as described below. The statistics were produced at CRN, which is statutory, so no consent was required according to the Norwegian Health Register Act §19.

### Incidence

We reported age-standardized incidence rates (ASIR) of histologically verified gliomas per 100,000 person-years for each histopathology group overall, and by sex and age groups (child: 0–17; young adult 18–39; adult 40–69; and older adult 70–99 years). Incidence rates were directly age-standardized to the age-distribution of the Norwegian population in 2021. To compare incidence patterns by histopathology group, we plotted a Lowess smooth (bandwidth 0.8) of incidence over age (0–9, 10–19, …, 80–99 years), and a 5-year rolling average of ASIR over time (2002–2006, 2003–2007, …, 2017–2021).

### Survival

We used RS for estimation of survival probability at certain time points from diagnosis. RS is calculated by dividing the observed survival probability of the study population by the expected survival probability of the general population of the same attained age, sex, calendar year and residency. RS allows a fair comparison of survival between patient groups that are not comparable, for example in terms of age-distribution.

We estimated RS up to 10 years from diagnosis using the non-parametric Pohar Perme estimator [16] for each histopathology group overall, and by sex, age group and diagnosis period. RS was age-standardized using the Brenner approach [17, 18], weighted to the age distribution of all patients diagnosed with histologically verified glioma during the years 2017–2021 (age-weight terciles: 0–48, 49–65, 66–99 years). To estimate RS, we required (1) at least 30 cases at the start of follow-up, and (2) 10 cases remaining at the time point of estimation (e.g. 5 years after diagnosis). Age-standardization of RS additionally required (3) at least three cases in each age-weight tercile at the start of follow-up. The histopathology groups pilocytic astrocytoma and unique astrocytoma variant did not meet criterion (3), so crude RS probabilities were reported instead. We used likelihood ratio tests to look for significant differences in survival by age group or diagnosis period in Cox models adjusted for age and period.

We further evaluated survival trends by plotting age-standardized 1- and 5-year RS for 5-year rolling diagnosis periods, from 2002–2006 (labelled 2006) to 2017–2021 (labelled 2021). A Wald test was used to test for linear trend in 5-year RS. For periods where all patients had 5 years of potential follow-up (until 2013–2017), we used a standard cohort method to estimate RS. For periods where some patients had less than 5 years of potential follow-up (i.e. periods including patients diagnosed in 2018 or later), we used a period analysis to estimate RS where survival time was borrowed from diagnosis years preceding the estimation period [19]. We tested for significant trend in 5-year RS in a linear regression model.

In supplementary analyses, we plotted Kaplan-Meier estimates of overall survival for histopathology groups stratified by sex, age group and diagnosis period. We tested for association between covariates and overall survival using log-rank tests. We further estimated median survival for histopathology groups by age and period.

## Results

### Incidence

Between 2002 and 2021, 7,048 new gliomas were histopathologically diagnosed in Norway, with an overall ASIR of 7.4 (95% CI: 7.3–7.6) per 100,000 person-years. Of these, 6,271 (89%) cases were categorized as malignant (ASIR 6.7 per 100,000, 95% CI: 6.5–6.9) and 777 (11%) as non-malignant (ASIR 0.8 per 100,000, 95% CI: 0.7–0.8). Incidence rates were generally higher for males (ASIR 8.8 per 100,000; 95% CI: 8.5–9.1) than females (ASIR 6.1 per 100,000; 95% CI: 5.9–6.4) with an overall ratio of 1.4 to 1. Glioblastoma was most common (55% of cases), followed by diffuse astrocytoma WHO grade II (8%) and anaplastic astrocytoma WHO grade III (7%). Further descriptive epidemiology of ASIR by histopathology group, sex, and age at diagnosis is outlined in Supplementary Table 4.

**Figure 1:**
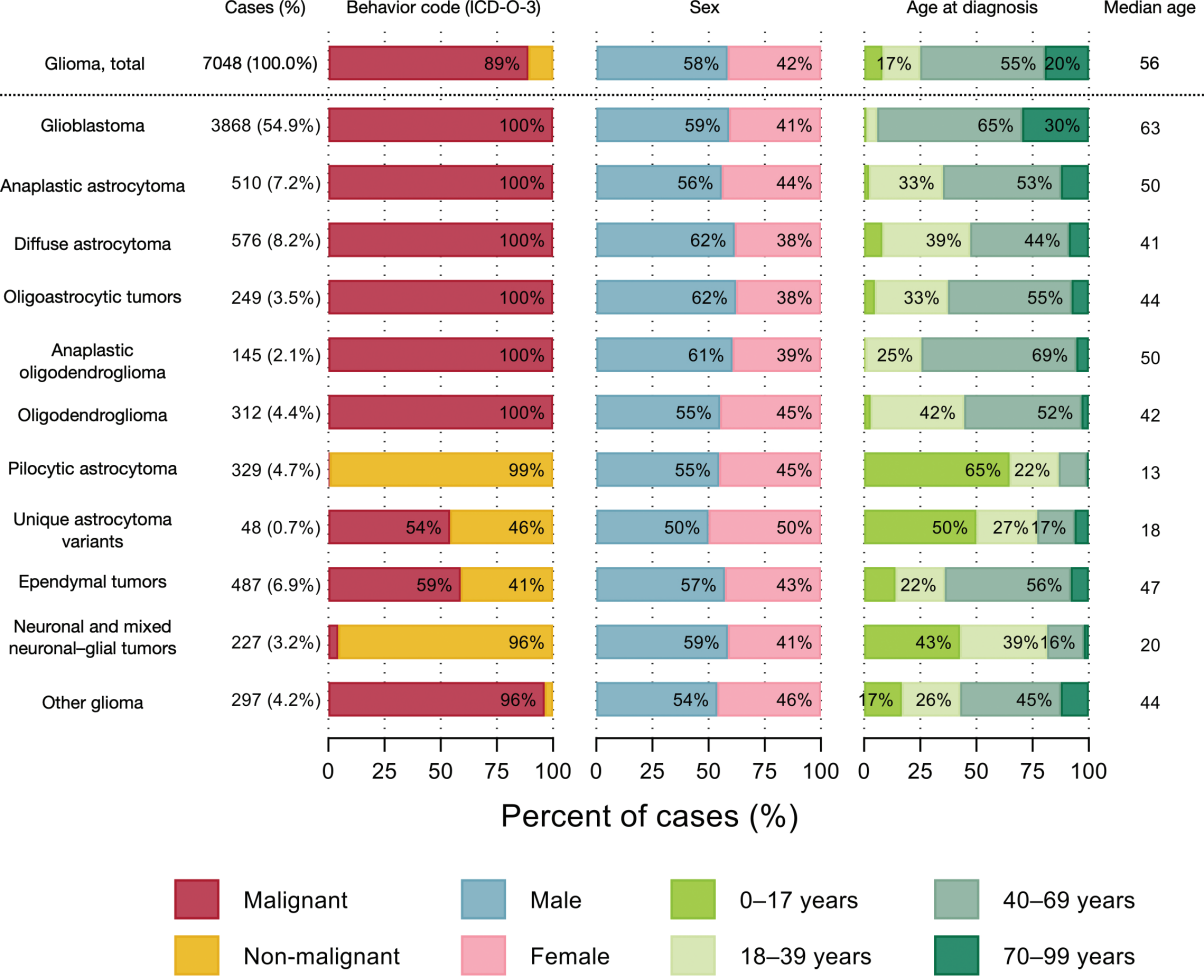
Number and percent of cases, by histopathology group, ICD-O-3 behavior code (malignant vs. non-malignant), sex, and age at diagnosis. Patients with histologically verified gliomas diagnosed during 2002–2021 (N = 7048).

The predominant histopathology group varied considerably by age at diagnosis (Figure 1). In children (0–17 years), pilocytic astrocytoma was most common (ASIR 1.0 per 100,000, 95% CI: 0.8–1.1), with a median age of 13 years at diagnosis. In adults (≥ 18 years), glioblastoma was most common (ASIR 5.3 per 100,000 person-years, 95% CI: 5.1–5.4), with a median age of 63 years. The distribution of ICD-O-3 behavior code (malignant vs. non-malignant), sex, and age at diagnosis for histopathology groups are depicted in Figure 1.

**Figure 2:**
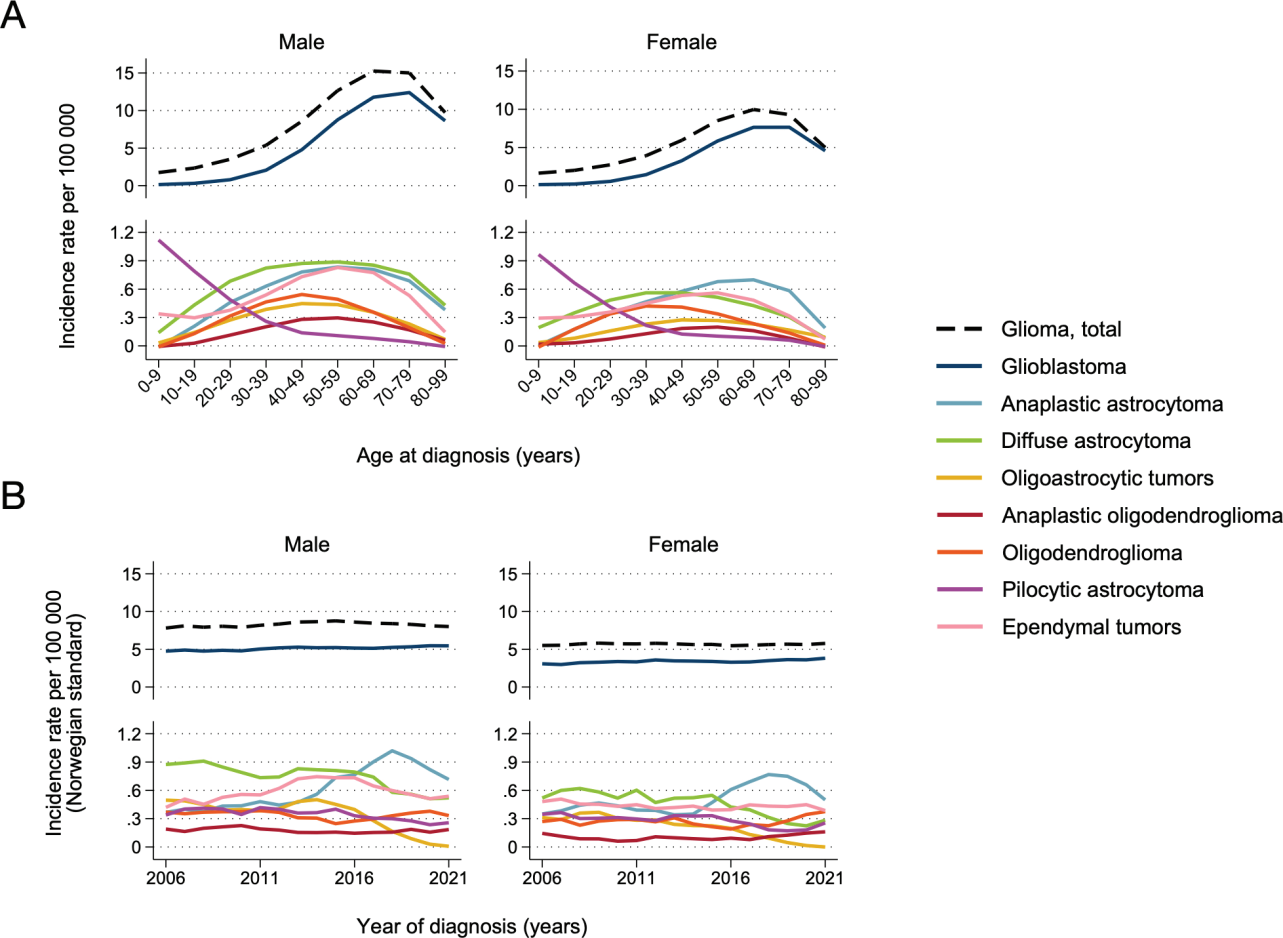
Incidence rate per 100,000 person-years for selected histopathology groups, by age (A) and year of diagnosis (B). Patients with histologically verified gliomas diagnosed during 2002–2021 (*N* = 6,475). Panel A shows a Lowess smooth of age-specific incidence rates. Panel B shows a 5-year rolling average of age-standardised incidence rates (ASIR) standardised to the 2021 Norwegian population.

The incidence rates by age followed three patterns: (1) increasing with age (glioblastoma), (2) decreasing with age (pilocytic astrocytoma), and (3) distribution along an inverted U-shaped curve (all other histopathology groups, Figure 2A).

Overall incidence was stable over time (Figure 2B). Glioblastoma incidence increased somewhat over time from ASIR 4.8 to 5.5 for men and 2.7 to 3.8 cases per 100,000 person-years for women between 2006 and 2021 (Figure 2B). Oligodendroglial tumor incidence was stable. For other astrocytoma subgroups in adults, the incidence trends over time were influenced by the changed WHO classification in 2016; for example continued use of the histopathology group oligoastrocytic tumors was strongly discouraged [20]. This subsequently altered the incidence rates of the subtypes of non-glioblastoma astrocytic tumors, exemplified by a rapid decrease in the number of oligoastrocytic tumors and corresponding increase in the number of anaplastic astrocytoma after 2016 (Figure 2B).

### Survival

**Figure 3:**
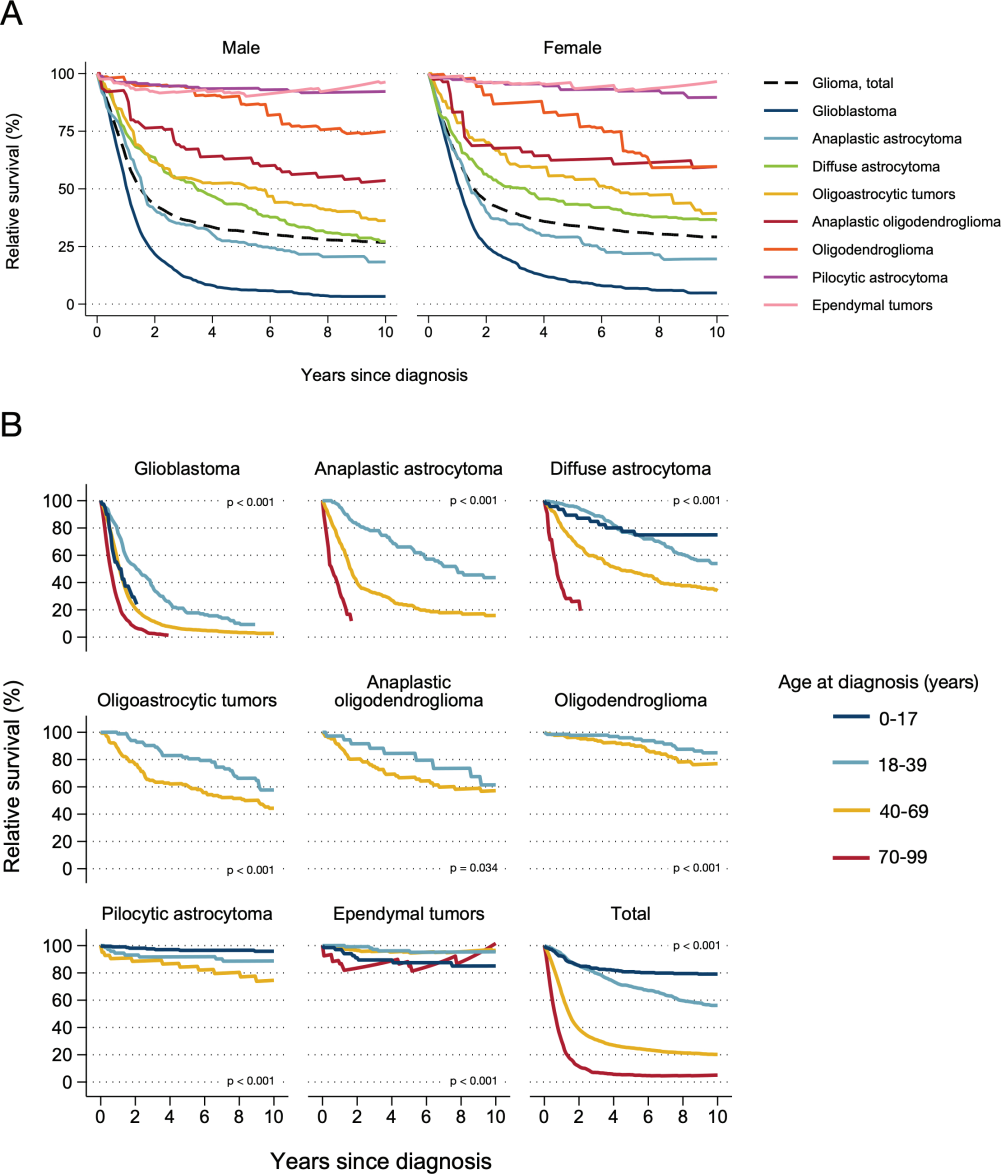
Age-specific relative survival up to 10 years from diagnosis for selected histopathology groups, by sex (A) and age-group (B). Patients diagnosed with histologically verified gliomas during 2002–2021 (*N* = 6475). Pohar Perme estimates of relative survival were age-standardized to the distribution of all patients diagnosed with histologically verified gliomas during 2017–2021. Pilocytic astrocytoma, most common among children, did not meet the criteria for age-standardization, so crude relative survival was estimated. P-values represent age group comparisons.

Overall, RS after 1-, 5- and 10-years, respectively, was 63.6% (95% CI: 62.5–64.8%), 32.8% (31.6–33.9%) and 27.9% (26.7–29.1%). Although not statistically significant (p-value: 0.062), the RS was slightly higher for females than males (Table 1). Survival probability varied, however, widely by histopathology group and age (Table 2, Figure 3, overall survival in Supplementary Figure 2 and 3). The lowest 5-year RS was found in adults with glioblastoma (7.6%, 95% CI: 6.5–8.8%) and the highest for patients with ependymal tumors (92.4%, 95% CI: 88.2–96.8%, age-standardized RS) and pilocytic astrocytoma (93.4%, 95% CI: 90.5–96.3%, crude RS). Within the histopathology groups where all tumors were classified with ICD-O-3 malignant behavior code (glioblastoma, diffuse and anaplastic astrocytoma, oligoastrocytic tumors, oligodendroglioma and anaplastic oligodendroglioma), we further found a wide range of 5-year survival probabilities; lowest for glioblastoma (above) and highest for oligodendroglioma (84.9%, 95% CI: 76.2–94.5%). Except for oligodendroglioma, 5- and 10-year survival probabilities were slightly higher for adult women than men (Table 1). There was also a statistically significant (*p* < 0.001) association between poorer survival with increasing age at diagnosis across all histopathology groups (Table 2, Figure 3B). For example, 1-year RS for the histopathology groups where all tumors were classified with malignant behavior code (except glioblastoma) was > 95% in young adults (18–39 years) and < 40% in older adults (≥ 70 years).

**Table 1:** Age-standardized relative survival (RS) with 95% confidence intervals (CI), by histopathology group and sex, for patients with histologically verified gliomas diagnosed during 2002–2021 (*N* = 7,048). Non-parametric Pohar Perme estimates of RS were age-standardized (Brenner approach) to the age distribution of all patients diagnosed with histologically verified gliomas during 2017–2021. Relative survival was estimated up to 10 years from diagnosis if there were at least 30 patients at the start of follow-up and at least 10 patients remaining at the time of estimation. Age-standardization additionally required at least three patients in each age-weight tercile.

Histopathology group	*N*	Years from diagnosis	Total RS (%) (95% CI)	Males RS (%) (95% CI)	Females RS (%) (95% CI)
Glioma, total	7048	1	63.6 (62.5–64.8)	63.6 (62.1–65.1)	63.7 (62.0–65.6)
		5	32.8 (31.6–33.9)	31.8 (30.3–33.4)	34.1 (32.3–36.0)
		10	27.9 (26.7–29.1)	27.0 (25.5–28.6)	29.2 (27.4–31.1)
Glioblastoma	3868	1	51.8 (50.1–53.6)	51.3 (49.1–53.6)	52.6 (50.0–55.3)
		5	7.6 (6.5–8.8)	6.2 (4.9–7.7)	9.7 (7.9–11.9)
		10	4.0 (3.0–5.1)	3.3 (2.3–4.8)	4.8 (3.3–7.1)
Anaplastic astrocytoma	510	1	66.4 (61.8–71.4)	69.0 (62.6–76.0)	63.6 (57.0–71.0)
		5	27.8 (23.9–32.5)	26.8 (21.6–33.3)	29.1 (23.4–36.3)
		10	18.9 (15.0–23.8)	18.3 (13.2–25.4)	19.6 (14.1–27.2)
Diffuse astrocytoma	576	1	72.9 (67.9–78.3)	74.2 (68.1–80.9)	70.5 (62.0–80.2)
		5	43.4 (38.2–49.2)	43.4 (37.1–50.7)	43.3 (35.0–53.6)
		10	30.6 (25.8–36.2)	27.2 (21.4–34.6)	36.3 (28.7–45.9)
Oligoastrocytic tumor	249	1	79.5 (71.6–88.3)	79.9 (69.5–91.9)	78.6 (66.9–92.3)
		5	52.9 (44.5–63.0)	52.0 (41.9–64.6)	55.2 (41.9–72.8)
		10	37.2 (29.5–47.0)	36.2 (26.9–48.7)	39.4 (27.5–56.4)
Anaplastic oligodendroglioma	145	1	88.1 (80.3–96.5)	90.9 (82.2–100.6)	83.3 (68.4–101.5)
		5	62.3 (51.4–75.4)	63.2 (50.2–79.6)	62.7 (45.6–86.1)
		10	55.2 (44.2–68.8)	53.6 (40.6–70.8)	59.6 (42.3–84.2)
Oligodendroglioma	312	1	97.0 (93.6–100.6)	95.9 (90.4–101.7)	98.8 (96.3–101.4)
		5	84.9 (76.2–94.5)	86.7 (76.5–98.2)	82.2 (67.9–99.7)
		10	68.7 (57.1–82.7)	74.9 (61.3–91.5)	59.7 (41.8–85.3)
Pilocytic astrocytoma^*^	329	1	96.7 (94.8–98.7)	96.2 (93.4–99.1)	97.4 (94.8–100.0)
		5	93.4 (90.5–96.3)	93.6 (89.9–97.5)	93.1 (88.7–97.7)
		10	91.1 (87.5–94.7)	92.2 (87.9–96.8)	89.7 (84.1–95.7)
Unique astrocytoma variant^*^	48	1	91.7 (84.2–100.0)	<30 at start	<30 at start
		5	75.1 (63.6–88.6)		
		10	66.8 (53.9–82.8)		
Ependymal tumor	487	1	96.3 (93.7–99.1)	94.9 (91.2–98.8)	98.8 (95.8–101.9)
		5	92.4 (88.2–96.8)	91.5 (86.0–97.4)	94.1 (87.8–100.8)
		10	96.4 (90.9–102.3)	96.3 (89.1–104.1)	96.5 (88.5–105.4)
Neuronal and mixed neuronal-glial tumor	227	1	81.3 (66.2–99.8)	73.4 (54.3–99.4)	**
		5	69.9 (53.8–90.9)	69.8 (50.3–96.8)	
		10	70.8 (54.5–92.1)	71.1 (51.2–98.6)	
Other glioma	297	1	67.2 (60.9–74.1)	64.4 (55.4–75.0)	70.3 (62.0–79.8)
		5	43.0 (36.6–50.4)	42.5 (33.7–53.7)	44.1 (35.4–54.9)
		10	37.3 (30.9–45.1)	35.6 (26.4–48.0)	39.3 (30.7–50.4)

* Histopathology groups that were most common among children did not meet the criteria for age-standardization, so crude relative survival estimates are shown instead.

** Did not meet criteria of 3 cases per age group for standardization.

**Table 2:** Age-specific relative survival (RS) with 95% confidence intervals (CI), by histopathology group and age at diagnosis, for patients with histologically verified gliomas diagnosed during 2002–2021 (*N* = 7048). Non-parametric Pohar Perme estimates of RS were age-standardized (Brenner approach) to the age distribution of all patients diagnosed with histologically verified gliomas during 2017–2021. RS was estimated up to 10 years from diagnosis if there were at least 30 patients at the start of follow-up and at least 10 patients remaining at the time of estimation.

Histopathology group	Years from diagnosis	Child (0–17 years)	Young adult (18–39 years)	Adult (40–69 years)	Older adult (70–99 years)
		RS (%) (95% CI)	RS (%) (95% CI)	RS (%) (95% CI)	RS (%) (95% CI)
Glioma, total (*N* = 7,048)	1	92.3 (90.2–94.5)	94.0 (92.7–95.4)	65.7 (64.3–67.3)	29.7 (27.4–32.3)
	5	82.3 (79.2–85.6)	72.7 (70.1–75.5)	26.4 (25.0–27.9)	5.4 (4.2–7.0)
	10	80.4 (77.0–83.9)	59.7 (56.6–63.0)	21.6 (20.2–23.2)	4.9 (3.5–6.9)
Glioblastoma (N = 3,868)	1	52.5 (39.1–70.5)	76.2 (70.3–82.6)	54.3 (52.4–56.3)	26.2 (23.7–28.9)
	5	<10 left	17.8 (12.8–24.7)	5.7 (4.8–6.8)	<10 left
	10		<10 left	2.8 (2.0–3.8)	
Anaplastic astrocytoma (*N* = 510)	1	<30 at start	95.3 (92.1–98.6)	69.4 (64.0–75.2)	32.7 (22.8–46.9)
	5		66.1 (58.8–74.3)	23.2 (18.4–29.3)	<10 left
	10		43.7 (34.4–55.5)	15.9 (11.1–22.6)	
Diffuse astrocytoma (*N* = 576)	1	93.6 (86.9–100.9)	97.8 (95.9–99.8)	80.6 (75.8–85.7)	36.3 (25.0–52.7)
	5	77.6 (66.1–91.0)	76.4 (70.7–82.6)	48.5 (42.5–55.4)	<10 left
	10	75.0 (63.0–89.2)	54.0 (46.9–62.1)	34.0 (27.9–41.4)	
Oligoastrocytic tumor (*N* = 249)	1	<30 at start	98.8 (96.5–101.3)	87.9 (82.6–93.7)	<30 at start
	5		81.8 (73.7–90.7)	60.9 (53.0–69.9)	
	10		57.7 (47.2–70.6)	44.3 (36.1–54.4)	
Anaplastic oligodendroglioma (*N* = 145)	1	<30 at start	97.3 (92.1–102.8)	89.4 (83.4–95.8)	<30 at start
	5		84.6 (72.6–98.5)	66.9 (57.5–77.8)	
	10		61.5 (43.1–87.8)	57.2 (46.3–70.8)	
Oligodendroglioma (*N* = 312)	1	<30 at start	98.5 (96.4–100.7)	97.9 (95.6–100.3)	<30 at start
	5		96.0 (92.3–99.8)	90.6 (85.5–96.0)	
	10		85.0 (77.6–93.1)	77.0 (68.9–86.1)	
Pilocytic astrocytoma^*^ (*N* = 329)	1	99.1 (97.8–100.4)	94.5 (89.3–99.9)	90.6 (81.9–100.2)	<30 at start
	5	96.5 (93.9–99.2)	91.8 (85.5–98.5)	84.5 (73.1–97.6)	
	10	95.9 (92.9–98.9)	88.7 (81.3–96.8)	74.6 (59.9–92.8)	
Unique astrocytoma variant^*^ (*N* = 48)	1	<30 at start	<30 at start	<30 at start	<30 at start
	5				
	10				
Ependymal tumor (*N* = 487)	1	97.1 (93.1–101.2)	100.1 (100.1–100.1)	98.6 (97.0–100.2)	86.6 (75.8–99.1)
	5	87.5 (79.6–96.1)	96.2 (92.4–100.3)	95.0 (91.7–98.4)	84.9 (69.7–103.4)
	10	85.1 (76.2–95.0)	95.5 (91.1–100.1)	96.6 (92.5–100.8)	101.6 (78.5–131.5)
Neuronal and mixed neuronal-glial tumor (*N* = 227)	1	99.0 (97.0–101.0)	98.9 (96.7–101.2)	97.6 (92.5–103.0)	<30 at start
	5	96.6 (92.7–100.6)	97.7 (94.2–101.4)	90.1 (79.7–101.8)	
	10	96.7 (92.8–100.8)	96.5 (91.9–101.3)	87.2 (73.6–103.2)	
Other glioma (*N* = 297)	1	76.0 (65.1–88.8)	94.9 (90.0–100.0)	78.6 (71.8–86.0)	22.3 (12.1–41.3)
	5	62.1 (50.0–77.1)	77.1 (68.0–87.5)	44.9 (36.9–54.7)	<10 left
	10	59.7 (47.4–75.2)	62.3 (51.4–75.4)	41.8 (33.6–52.1)	

* Histopathology groups that were most common among children did not meet the criteria for age-standardization, so crude relative survival estimates are shown instead.

**Figure 4:**
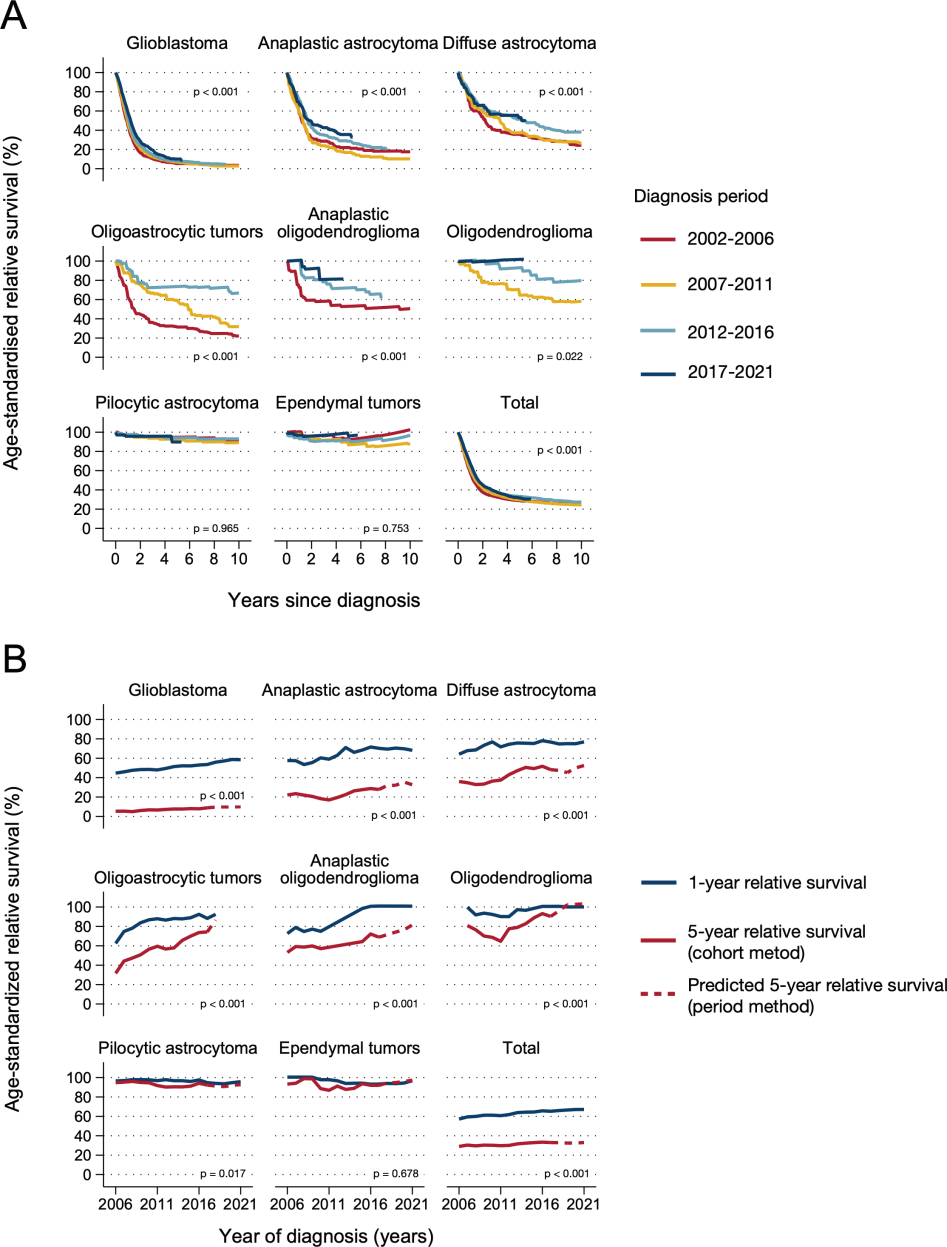
Age-standardized relative survival for selected histopathology groups, by period (A) and year (B) of 1-year and 5-year relative survival (5-year rolling average). Histologically verified gliomas diagnosed during 2002–2021 (*N* = 6,475). Panel A shows p-values for trends over time periods. Panel B shows p-values for trend in 5-year relative survival over time.

Over time, RS improved significantly for glioma patients overall (*p* < 0.001) and for most histopathology groups (Figure 4A). The crude overall survival did not, however, improve significantly over time (p = 0.36, Supplementary Figure 4), probably because absolute survival estimates failed to adjust for the larger fraction of glioblastoma patients (who are also older) over time. For example, during 2002–2006 glioblastoma represented 50% of all gliomas, compared to 61% in 2017–2021. Patients 70 years or older comprised 24% of patients in 2017–2021, compared to 17% in 2002–2006.

**Figure 5:**
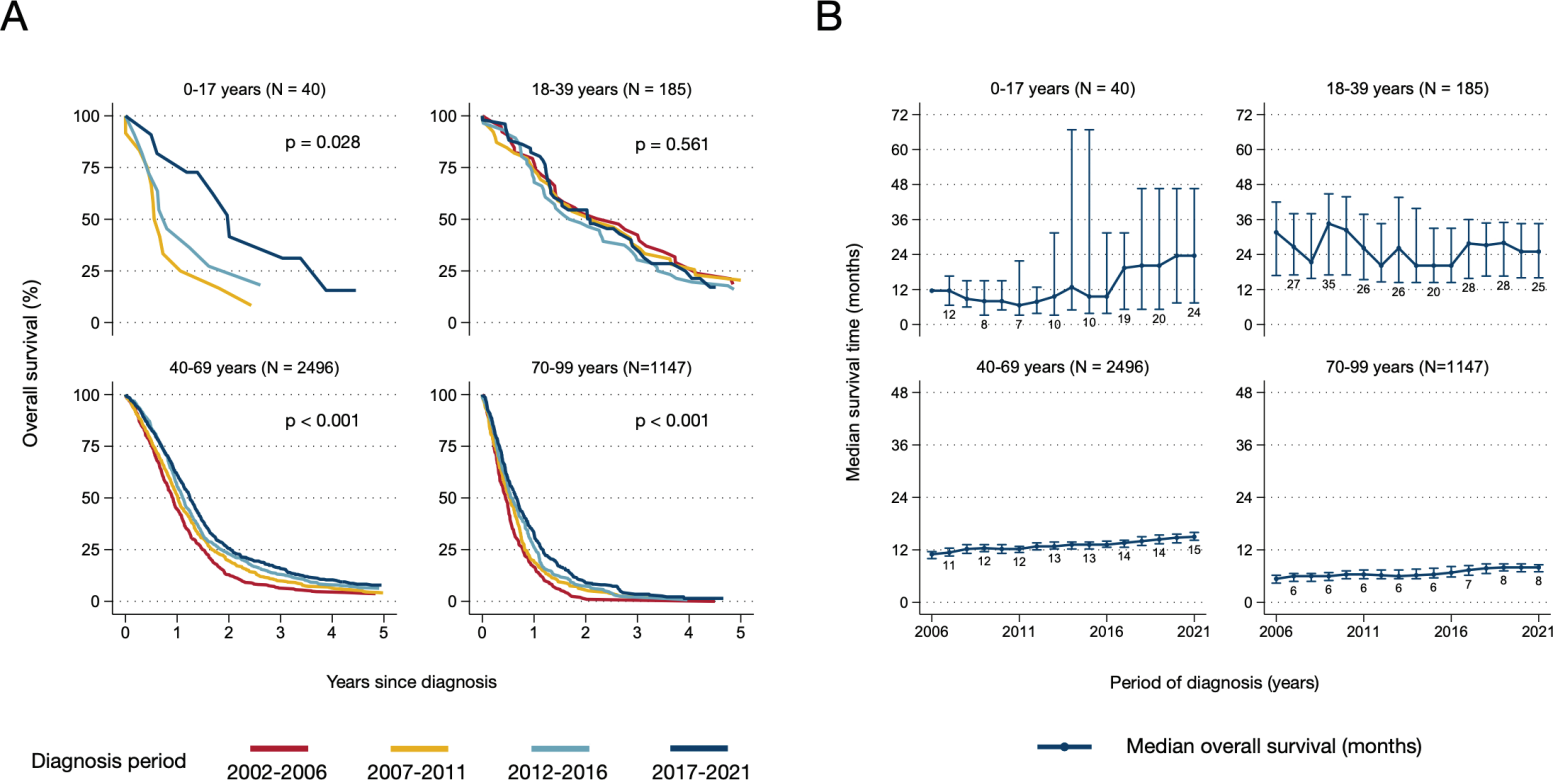
Trends in survival of glioblastoma patients by age group and year of diagnosis. (A) Kaplan-Meier estimates of overall survival up to 5 years from diagnosis, by age and period of diagnosis. (B) Median overall survival with 95% confidence intervals, by age and year of diagnosis (5-year rolling average). Patients diagnosed with histologically verified glioblastomas during 2002–2021 (*N* = 3,868). P-values represent period comparison.

Over 20 years, 1- and 5- year RS became progressively higher for most glioma histopathology groups; 1- and 5-year RS improved from 57 and 29% in 2001–2006 to 67 and 33% in 2017–2021, respectively (Figure 4B). Notably, RS increased within all histopathological groups where all tumors were classified with malignant behavior code (Figure 4B). Even glioblastoma patients had clinically and statistically significant improvements in 1- and 5-year RS over time (1-year RS from 44.6% in 2006 to 58.5% in 2021, 5-year RS from 5.4 to 10.0% between 2006 and 2021, *p* < 0.001, Figure 4B). Crude overall survival of glioblastoma patients also improved over time for adults and older adults (40–69 and 70+ years, both *p* < 0.001, Figure 5A). For adults 40–69 years, median survival improved from 11 to 15 months between 2006 and 2021, and 5-year overall survival improved from 3.7% (95% CI: 2.3–5.6%) to 6.3% (95% CI: 4.6–8.4%). Even for the oldest glioblastoma patients (70+ years), median survival improved from 6 to 8 months between 2006 and 2021, with a corresponding increase in 1-year overall survival from 16.8% (95% CI: 12.1–22.2%) to 32.8% (95% CI: 28.3–37.4%, Figure 5B). An overview of overall survival estimates across all histopathology groups by age and period is outlined in Supplementary Table 5.

## Discussion

We found that glioma incidence has been stable for men and women over time, that incidence by histopathology group followed established age-specific distributions, and that 1- and 5-year RS of patients with malignant glioma has slowly increased in Norway, also for older glioblastoma patients (70+ years). We further confirmed established knowledge; glioblastoma was the most common histological subtype; there was a male predominance in diagnosed glioma; females had somewhat better survival than males, and age was an important prognostic factor across all malignant glioma histopathological groups.

We found that the incidence rate of histologically verified gliomas was similar to rates in recent reports from other Nordic countries [4, 5] and Canada [21], where the populations’ age-distributions were comparable to Norway. However, the age-standardized incidence was higher than several other large epidemiological cohorts from Europe [6, 7] and the US [15], where the standard population was younger than in Norway. Comparing incidence rates of glioma across countries and cancer registries is, however, not straightforward. The number of cases and the precision in reporting may be influenced by sources and completeness of the tracked data (national vs. regional cancer registries vs. health record systems) [21], whether reporting includes children and adults, variation in the proportion of histologically verified tumors [22–24], and the reference population used for age-standardization (World, US vs. European Standard Population) [4, 5, 15]. Also, the definition of glioma is not universal. For example, we have included the ICD-O-3 codes 9505 (ganglioglioma) and 9509 (glioneural tumor) (*n* = 147, 2% of tumors in this study), while these are omitted by CBTRUS [15]. Collectively, such factors may hamper direct comparison between reports, emphasizing the importance of providing continuous and up-to-date reporting of incidence and survival trends from cancer registries at the national level.

Dissecting variations at the histopathological group level revealed that the incidence rate of the most common subtype, glioblastoma, was higher in the CRN (4.2 per 100,000) than in the CBTRUS (3.27 per 100,000, 95% CI: 3.24–3.29) [15], but comparable to recent national registry reports from the Finnish, Danish and Canadian populations (ASIR from 3.5 to 5.1/100,000) [4, 5, 21]. Glioblastoma is typically diagnosed in elderly patients (median age at diagnosis 63 years in this study), and the incidence increases with age. A higher incidence rate in the Nordic region may be attributed to a high life expectancy, high completeness of reported cases, and universal health care allowing broad access to neuroimaging and invasive tissue sampling for verification of the diagnosis, even at an advanced age. In a recent report from the largest health region in Norway, only 5.5% of all glioblastoma patients did not have a tissue-based diagnosis during 2012–2017 [10]. In the present study, only 9.2% of glioblastomas registered by the CRN were clinically reported cases. The fraction of a non-tissue-based diagnoses is considerably higher (up to 25%) in selected reports from for example the US [22]. For the other histopathology groups in which we used the same classification as the CBTRUS and Brain Tumor Registry of Canada (BTRC), Norwegian incidence rates were comparable to other reports [15, 21].

In this study, we have described incidence patterns over 20 years, and found that the overall incidence was stable throughout the entire period, in agreement with several other reports from Europe and the US [5, 8]. Dissecting time trends for non-glioblastoma astrocytic and oligodendroglial tumors was more challenging because these CNS tumors were re-classified within the study period, in 2007 and 2016 [20]. For example, the discontinuation of the diagnostic entity oligoastrocytoma in the 2016 WHO classification most likely influenced the incidence rates of other astrocytic and oligodendroglial tumors from 2016 and onwards.

For glioblastoma, the incidence increased slightly over time. This finding agrees with reports from various countries [5, 8, 25, 26], and may be attributable to improved access to neuroimaging, invasive procedures for tissue-based diagnostics [26], and in other series (not standardized to the Norwegian 2021 population as in this study) an ageing population [25]. Additionally, the changed recommendation for diagnosis of molecular glioblastoma by the cIMPACT-NOW initiative in 2018 [27] likely influenced the incidence rates of anaplastic astrocytoma (decrease) and glioblastoma (increase) from 2018 and onwards.

Gliomas are typically associated with poor prognoses, with around two-thirds of patients surviving 1 year and just one-third surviving 5 years after diagnosis. The variation between entities was, however, extensive. For example, less than one in 10 glioblastoma patients survived 5 years from diagnosis compared to more than nine in 10 with ependymal tumors. The RS of the various histopathological groups in this study was comparable to other reports, but slightly lower than in the CBTRUS [15], and slightly higher than in the BTRC [21]. However, similar to the comparison of incidence rates, it is not straightforward to directly compare survival probabilities across registries and countries, which may have variations in the ascertainment of the deaths and the reference population for age-standardization.

Interestingly, we found improved survival over time, with a 10% gain in 1-year and 4% gain in 5-year RS over two decades. Survival improved over time for all histopathology groups where all tumors were classified with malignant behavior code (glioblastoma, diffuse and anaplastic astrocytoma, oligoastrocytic tumors, oligodendroglioma and anaplastic oligodendroglioma). Notably, for glioblastoma, for which the current standard-of-care with the alkylating chemotherapy temozolomide was established almost two decades ago [28], we found a small improvement in survival over time, even for elderly patients (70+ years). In a previous Norwegian study using population data, an increased fraction of patients received more extensive surgical resection over this period, which could have contributed to improved survival [26]. During the study period, Norway has also established clinical care pathways (in 2014) for newly diagnosed brain tumors to secure timely diagnostics and treatment. However, whether this impacts survival is unknown. Nevertheless, similar improved survival trends for glioblastoma patients have been described in other Nordic countries and the US [8, 25].

The strengths of this study include the long time-period, the complete coverage of the Norwegian population, and the national universal health care system. The latter limits biases in epidemiological series such as regional differences, variations in health-seeking behavior, and cost-related incentives in health care [29]. Moreover, the CRN is recognized as having high-quality and prospectively recorded data which reduces information bias [12]. Collectively, this strengthens the present study to potentially represent a reference on glioma incidence and patient survival for future population-based studies in Norway and other countries with similar health care services and population age-distribution. On the other hand, we included only histologically verified cases, and omitted radiologically and post-mortem diagnosed cases. The true incidence may be slightly underestimated in the oldest age groups, and true survival correspondingly overestimated. Also, we did not have data on all anti-neoplastic treatments, which is an important prognostic factor for survival. Further, we report on epidemiological trends over a period that span new CNS-tumor classification [20]. This represents a challenge to create rational groups of gliomas. In this study, we selected histopathological groups in accordance with CBTRUS [15] to preserve a certain level of detail while also allowing for comparisons to other registry reports. We acknowledge, however, that this grouping is not standardized across registry reports from various epidemiological cohorts and countries [4, 5, 9].

The variation in incidence and survival by tumor type, sex, and age reflects the heterogeneity of gliomas. The small number of cases per year for many tumor types underscores the need to compile information across larger populations to survey national and regional differences in incidence and survival. The CRN provides up-to-date reporting on gliomas, while benign tumors such as meningiomas are historically underreported if there is no tissue-based diagnosis. This has led to the foundation of a new clinical quality register for primary brain and spinal cord tumors registered within the CRN. All neuropathological, neurosurgical, and oncological units involved in diagnostics and treatment of benign and malignant CNS tumors contribute data. In turn, this aims to generate high-quality data on the total burden of primary CNS neoplasms with a considerable level of detail at the national, regional, hospital and single patient-level.

In summary, population-level surveillance of gliomas in Norway over the past 20 years shows that incidence has been stable, whereas survival has increased slightly over time. Gliomas represent a heterogeneous group of primary CNS neoplasms with highly variable incidence and survival patterns by histopathology group, age, and sex. This underscores the need for continous and up-to-date reporting from cancer registries to monitor disease burden and allocate adequate health care resources in a population.

## Supplementary Material

Stable glioma incidence and increased patient survival over the past two decades in Norway: a nationwide registry-based cohort studys

Stable glioma incidence and increased patient survival over the past two decades in Norway: a nationwide registry-based cohort studys

## Data Availability

The data generated and analyzed in this study is available from the Cancer Registry of Norway upon reasonable request.
