# Supplementary material for: Stable glioma incidence and increased patient survival over the past two decades in Norway: a nationwide registry-based cohort study
Source: Acta Oncol. 2024 Mar 19;63:24970. doi: 10.2340/1651-226X.2024.24970 (PMC11332456; doi:10.2340/1651-226X.2024.24970)
Supplement: Stable glioma incidence and increased patient survival over the past two decades in Norway: a nationwide registry-based cohort studys [file AO-63-24970-s1.pdf]

Supplementary material has been published as submitted. It has not been copyedited or typeset by Acta Oncologica.

Supplementary Figure 1

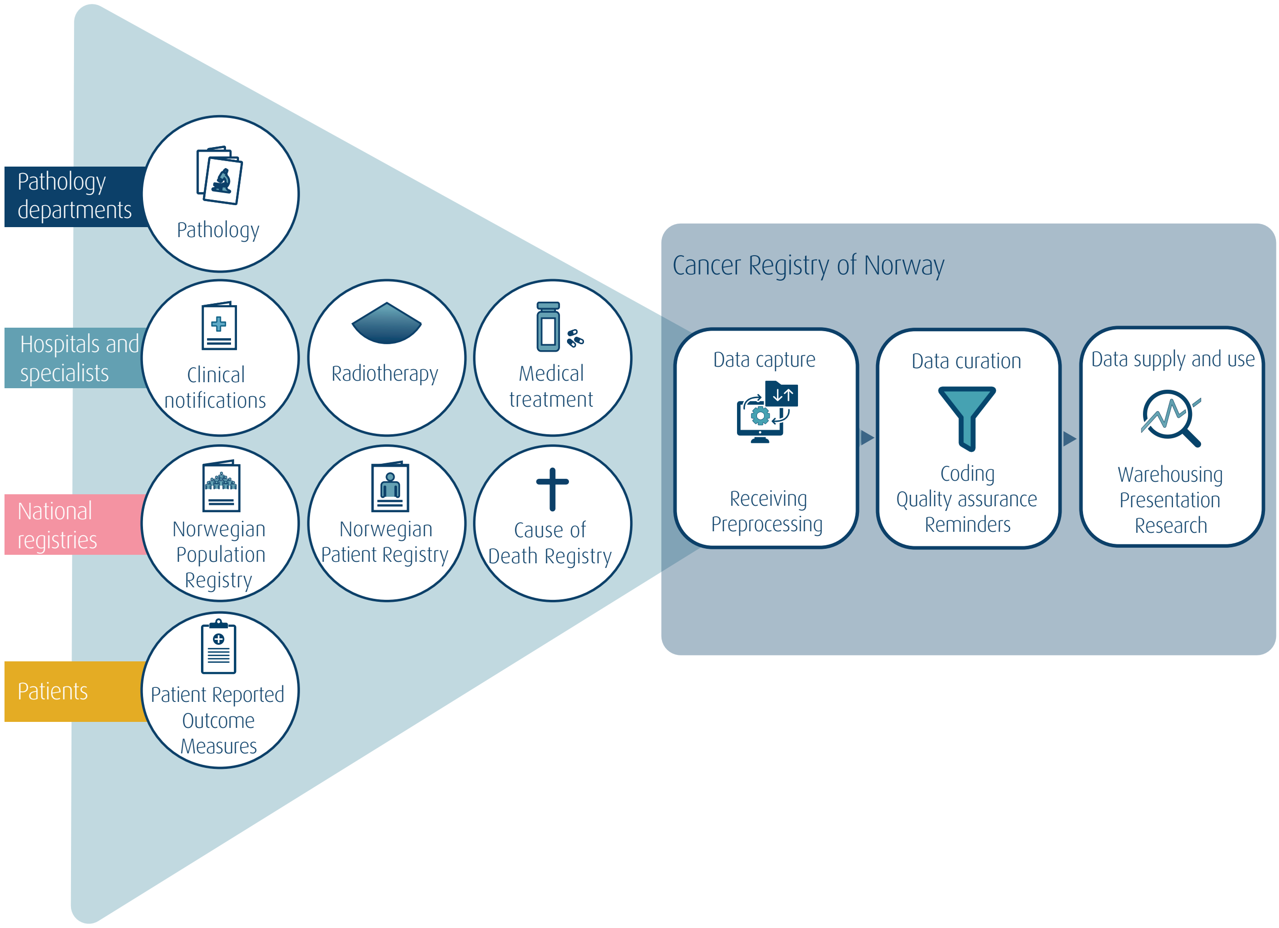

Supplementary Figure 1. Sources of information and the process of cancer registration at the Cancer Registry of Norway. Reproduced with permission from the most recent Cancer in Norway publication (Cancer Registry of Norway. Cancer in Norway 2022 - Cancer incidence, mortality, survival and prevalence in Norway. Oslo: Cancer Registry of Norway, 2023). Available through: https://www.kreftregisteret.no/globalassets/cancer-in-norway/2022/cin_report-2022.pdf.

Supplementary Figure 2

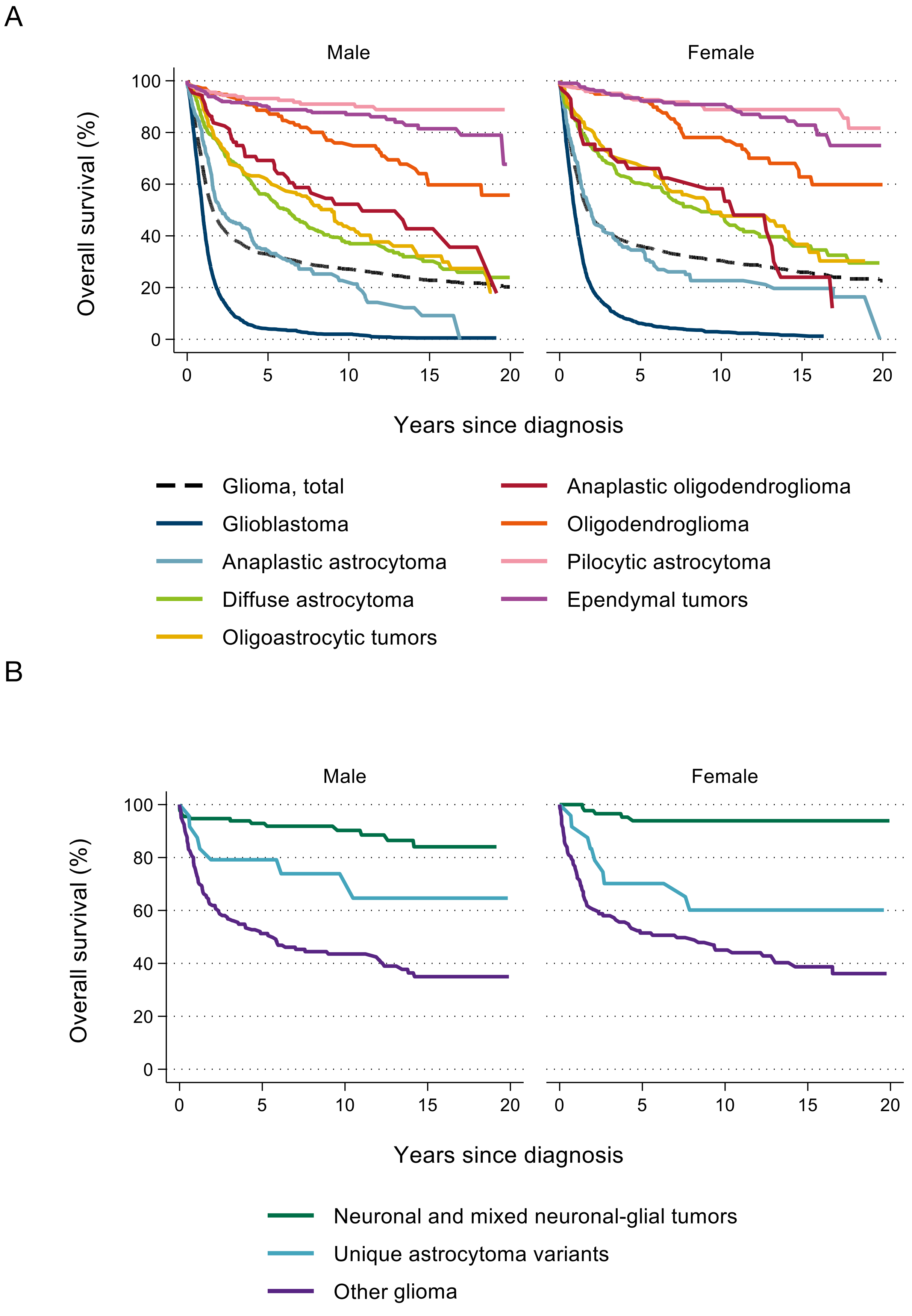

Supplementary Figure 2. Kaplan-Meier estimates of overall survival up to 20 years from diagnosis, by histopathology group and sex. Patients diagnosed with histologically verified gliomas during 2002—2021 (N=7048).

Supplementary Figure 3

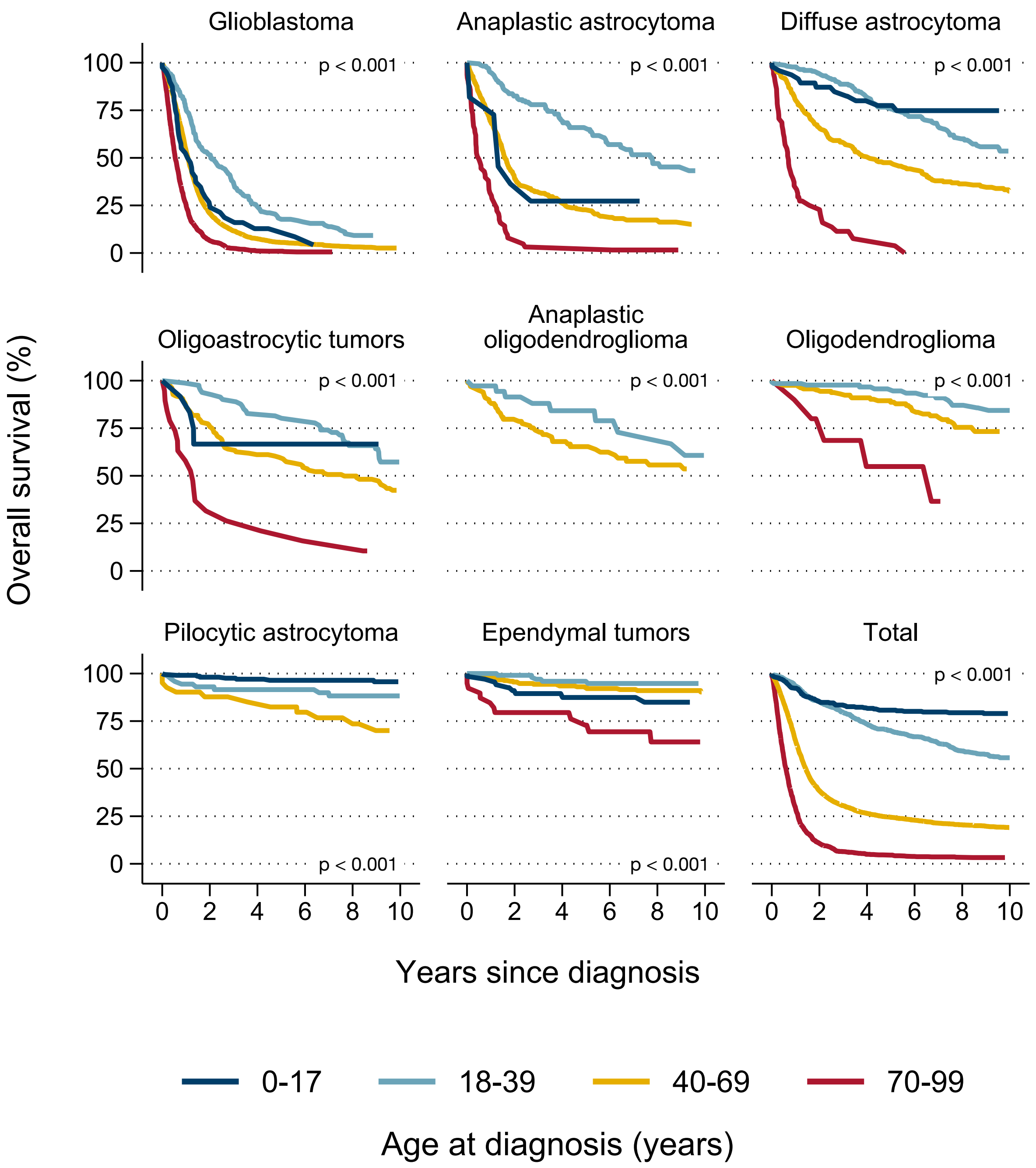

Supplementary Figure 3. Kaplan-Meier estimates of overall survival up to 10 years from diagnosis, by histopathology group and age at diagnosis. Patients diagnosed with histologically verified gliomas during 2002—2021 (N=7048). P-values represent age group comparison.

Supplementary Figure 4

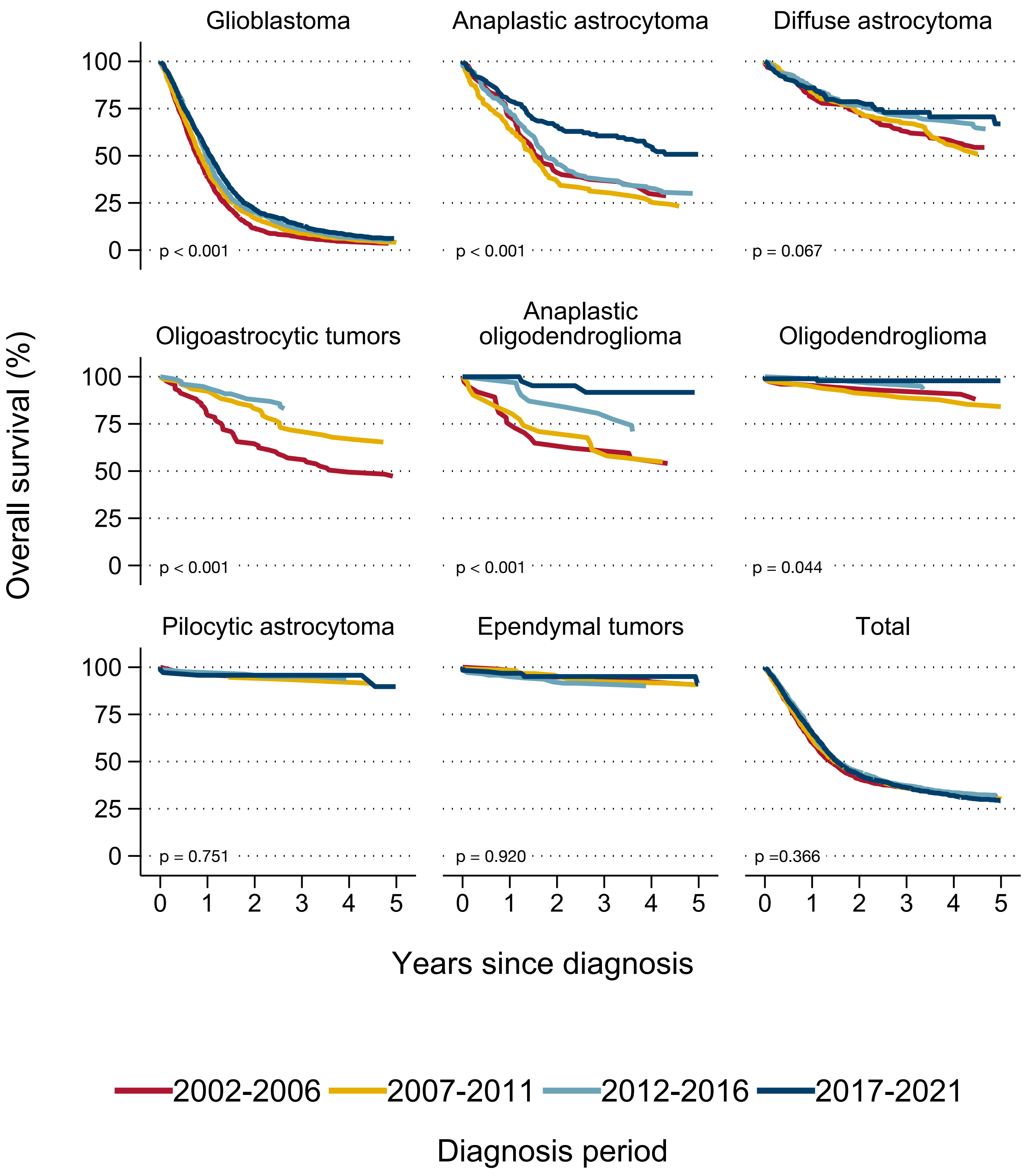

Supplementary Figure 4. Kaplan-Meier estimates of overall survival up to five years from diagnosis, by histopathology group and period of diagnosis. Patients diagnosed with histologically verified gliomas during 2002—2021 (N=7048). P-values represent period comparison.
