# Supplementary material for: Stable glioma incidence and increased patient survival over the past two decades in Norway: a nationwide registry-based cohort study
Source: Acta Oncol. 2024 Mar 19;63:24970. doi: 10.2340/1651-226X.2024.24970 (PMC11332456; doi:10.2340/1651-226X.2024.24970)
Supplement: Stable glioma incidence and increased patient survival over the past two decades in Norway: a nationwide registry-based cohort studys [file AO-63-24970-s2.pdf]

Supplementary material has been published as submitted. It has not been copyedited or typeset by Acta Oncologica.

Supplementary Table 1: Estimated completeness of gliomas reported to the Cancer Registry of Norway during 2002—2021 (N=7713).

Histopathology group	Total cases reported to CRN N (%)	Histologically verified cases N	Clinically reported cases N	Autopsy or death certificate only N	Estimated completeness in CRN %
Glioma, total	7713 (100.0)	7048	628	37	98.8
Glioblastoma	4245 (55.0)	3868	358	19	98.8
Anaplastic astrocytoma	516 (6.7)	510	3	3	99.9
Diffuse astrocytoma	611 (7.9)	576	32	3	99.4
Oligoastrocytic tumor	249 (3.2)	249	0	0	100.0
Anaplastic oligodendroglioma	145 (1.9)	145	0	0	100.0
Oligodendroglioma	314 (4.1)	312	2	0	99.9
Pilocytic astrocytoma	342 (4.4)	329	12	1	99.8
Unique astrocytoma variant	50 (0.6)	48	1	1	99.7
Ependymal tumor	532 (6.9)	487	39	6	98.4
Neuronal and mixed neuronal-glial tumor	249 (3.2)	227	19	3	98.5
Other gliomas	460 (6.0)	297	162	1	95.2

Supplementary Table 2: Number of glioma cases reported to the Cancer Registry of Norway, by histopathology group, morphology ICD-O-3 code, and data source, 2002—2021 (N=7713).

Histopathology group	Morphology ICD-O-3	Histologically verified cases[#]	Clinically reported cases	Autopsy or death certificate only
Glioblastoma	9440/3	3771	356	19
	9441/3	37	1	0
	9442/3	60	1	0
Anaplastic astrocytoma	9401/3	510	3	3
Diffuse astrocytoma	9381/3	5	7	2
	9400/3	322	24	1
	9411/3	50	0	0
	9420/3	199	1	0
Oligoastrocytic tumor	9382/3	249	0	0
Anaplastic oligodendroglioma	9451/3	144	0	0
	9460/3	1	0	0
Oligodendroglioma	9450/3	312	2	0
Pilocytic astrocytoma	9421/1	327	12	1
	9425/3	2	0	0
Uniqu astrocytoma variant	9384/1	22	1	1
	9424/3	26	0	0
Ependymal tumor	9383/1	73	27	4
	9391/3	236	11	2
	9392/3	51	0	0
	9394/1	127	1	0
Neuronal and mixed neuronal-glial tumor	9412/1	3	0	0
	9413/0	77	16	1
	9505/1	118	3	1
	9505/3	10	0	1
	9509/1	19	0	0
Other glioma	9380/1	11	0	0
	9380/3	281	162	1
	9385/3	3	0	0
	9430/3	2	0	0

Supplementary Table 3: Number of histologically verified gliomas per year by CBTRUS histotype group and morphology ICD-O-3 code (N = 7048).

CBTRUS histotype	Morphology ICD-O-3	Total, 2002-2021	2002	2003	2004	2005	2006	2007	2008
**Total**	**Total**	**7048**	**303**	**300**	**291**	**336**	**312**	**347**	**320**
**Glioblastoma**	**Total**	**3868**	**142**	**148**	**151**	**165**	**162**	**157**	**175**
	9440/3	3771	138	147	146	161	152	150	169
	9441/3	37	1	1	5	1	2	4	1
	9442/3	60	3			3	8	3	5
**Anaplastic astrocytoma**	**Total**	**510**	**14**	**19**	**12**	**13**	**22**	**21**	**21**
	9401/3	510	14	19	12	13	22	21	21
**Diffuse astrocytoma**	**Total**	**576**	**32**	**22**	**40**	**33**	**31**	**43**	**29**
	9381/3	5	1				1	1	
	9400/3	322	24	18	23	24	21	21	14
	9411/3	50		2	2	2	4	6	8
	9420/3	199	7	2	15	7	5	15	7
**Oligoastrocytic tumors**	**Total**	**249**	**22**	**11**	**20**	**23**	**17**	**18**	**15**
	9382/3	249	22	11	20	23	17	18	15
**Anaplastic oligodendroglioma**	**Total**	**145**	**10**	**8**	**4**	**7**	**8**	**4**	**9**
	9451/3	144	9	8	4	7	8	4	9
	9460/3	1	1						
**Oligodendroglioma**	**Total**	**312**	**15**	**19**	**13**	**17**	**12**	**16**	**13**
	9450/3	312	15	19	13	17	12	16	13
**Pilocytic astrocytoma**	**Total**	**329**	**14**	**21**	**12**	**20**	**17**	**25**	**16**
	9421/1	327	14	21	12	20	17	25	16
	9425/3	2							
**Unique astrocytoma variants**	**Total**	**48**	**3**	**3**	**2**		**1**	**3**	**2**
	9384/1	22	1	2			1	2	1
	9424/3	26	2	1	2			1	1
**Ependymal tumors**	**Total**	**487**	**11**	**24**	**19**	**29**	**19**	**24**	**13**
	9383/1	73	1	2	3	2	5	4	2
	9391/3	236	5	11	9	17	9	11	5

	9392/3	51	2	5	2		2		2
	9394/1	127	3	6	5	10	3	9	4
**Neuronal and mixed neuronal-glial tumors**	**Total**	**227**	**15**	**7**	**6**	**11**	**9**	**9**	**8**
	9412/1	3		1				1	
	9413/0	77	7	3	4	5	5	4	4
	9505/1	118	6	3	2	4	1	4	4
	9505/3	10	2			2	1		
	9509/1	19					2		
**Other gliomas**	**Total**	**297**	**25**	**18**	**12**	**18**	**14**	**27**	**19**
	9380/1	11	1		1	1		1	3
	9380/3	281	24	17	11	17	14	26	16
	9385/3	3							
	9430/3	2		1					

2009	2010	2011	2012	2013	2014	2015	2016	2017	2018	2019	2020	2021
**340**	**327**	**364**	**381**	**367**	**355**	**382**	**359**	**414**	**400**	**387**	**377**	**386**
**180**	**187**	**198**	**218**	**188**	**190**	**206**	**197**	**235**	**243**	**240**	**239**	**247**
176	181	192	210	183	184	202	196	230	240	236	233	245
1	4	3	4	3	2	1		1	1		1	1
3	2	3	4	2	4	3	1	4	2	4	5	1
**21**	**11**	**25**	**19**	**21**	**33**	**50**	**46**	**49**	**50**	**24**	**25**	**14**
21	11	25	19	21	33	50	46	49	50	24	25	14
**31**	**19**	**35**	**29**	**48**	**32**	**25**	**20**	**21**	**18**	**22**	**16**	**30**
			1	1								
17	15	23	19	32	8	9	11	7	8	4	3	21
6		5	3	3	2		1	1	2	3		
8	4	7	6	12	22	16	8	13	8	15	13	9
**14**	**17**	**14**	**23**	**21**	**16**	**12**	**5**		**1**			
14	17	14	23	21	16	12	5		1			
**6**	**6**	**6**	**8**	**5**	**5**	**6**	**7**	**7**	**10**	**11**	**5**	**13**
6	6	6	8	5	5	6	7	7	10	11	5	13
**18**	**20**	**15**	**12**	**12**	**11**	**9**	**16**	**21**	**16**	**22**	**21**	**14**
18	20	15	12	12	11	9	16	21	16	22	21	14
**13**	**14**	**25**	**21**	**18**	**13**	**21**	**9**	**14**	**9**	**9**	**16**	**22**
13	14	25	21	18	13	21	9	14	8	8	16	22
									1	1		
**2**	**2**	**1**	**5**	**2**	**2**	**3**	**6**	**6**	**4**			**1**
2	1		2	1	2		4	1	1			1
	1	1	3	1		3	2	5	3			
**28**	**31**	**23**	**27**	**28**	**34**	**27**	**26**	**25**	**22**	**30**	**25**	**22**
7	5	4	1	6	4	5	3	6	3	3	1	6
14	13	11	19	15	17	11	15	12	11	10	10	11

2	4	3	2	5	2	1	5	1	4	3	5	1
5	9	5	5	2	11	10	3	6	4	14	9	4
**6**	**7**	**11**	**11**	**11**	**13**	**13**	**14**	**19**	**14**	**16**	**14**	**13**
	1											
2	2	3	5	2	5	2	4	4	5	4	3	4
4	4	4	5	8	6	10	9	12	5	12	8	7
		1	1			1	1		1			
		3		1	2			3	3		3	2
**21**	**13**	**11**	**8**	**13**	**6**	**10**	**13**	**17**	**13**	**13**	**16**	**10**
2	1	1										
19	11	10	8	13	6	10	13	17	13	13	14	9
											2	1
	1											

Supplementary Table 4 :

Cases (N) and age-standardized incidence rates (ASIR) per 100,000 person-years (2021 Norwegian standard) with 95% confidence intervals (CI), by histopathology group, sex, and age at diagnosis, of histologically verified gliomas diagnosed during 2002–2021 (N=7048).

Histopathology group	Variable	Total	Male	Female	Child -(0–17 years)	Young adult (18–39 years)	Adult -(40–69 years)	Older adult -(70–99 years)
Glioma, total	N	7048	4118	2930	572	1182	3909	1385
	ASIR (95% CI)	7.4 (7.3–7.6)	8.8 (8.5–9.1)	6.1 (5.9–6.4)	2.6 (2.4–2.8)	4.0 (3.8–4.3)	10.8 (10.5–11.2)	13.1 (12.4–13.8)
Glioblastoma	N	3868	2287	1581	40	185	2496	1147
	ASIR (95% CI)	4.2 (4.1–4.4)	5.1 (4.9–5.3)	3.4 (3.2–3.6)	0.2 (0.1–0.2)	0.6 (0.5–0.7)	7.0 (6.7–7.3)	10.8 (10.2–11.4)
Anaplastic astrocytoma	N	510	285	225	11	168	268	63
	ASIR (95% CI)	0.5 (0.5–0.6)	0.6 (0.5–0.7)	0.5 (0.4–0.5)	0.1 (0.0–0.1)	0.6 (0.5–0.7)	0.7 (0.7–0.8)	0.6 (0.5–0.8)
Diffuse astrocytoma	N	576	355	221	47	225	253	51
	ASIR (95% CI)	0.6 (0.5–0.6)	0.7 (0.6–0.8)	0.5 (0.4–0.5)	0.2 (0.2–0.3)	0.8 (0.7–0.9)	0.7 (0.6–0.8)	0.5 (0.4–0.6)
Oligoastrocytic tumor	N	249	155	94	12	81	137	19
	ASIR (95% CI)	0.3 (0.2–0.3)	0.3 (0.3–0.4)	0.2 (0.2–0.2)	0.1 (0.0–0.1)	0.3 (0.2–0.3)	0.4 (0.3–0.4)	0.2 (0.1–0.3)
Anaplastic oligodendroglioma	N	145	88	57	<10	36	100	<10
	ASIR (95% CI)	0.1 (0.1–0.2)	0.2 (0.1–0.2)	0.1 (0.1–0.2)	0.0 (0.0–0.0)	0.1 (0.1–0.2)	0.3 (0.2–0.3)	0.1 (0.0–0.1)
Oligodendroglioma	N	312	172	140	<10	130	163	10
	ASIR (95% CI)	0.3 (0.3–0.4)	0.3 (0.3–0.4)	0.3 (0.2–0.3)	0.0 (0.0–0.1)	0.4 (0.4–0.5)	0.4 (0.4–0.5)	0.1 (0.0–0.2)
Pilocytic astrocytoma	N	329	180	149	213	72	41	<10
	ASIR (95% CI)	0.3 (0.3–0.3)	0.3 (0.3–0.4)	0.3 (0.2–0.3)	1.0 (0.8–1.1)	0.2 (0.2–0.3)	0.1 (0.1–0.1)	0.0 (0.0–0.1)
Unique astrocytoma variant	N	48	24	24	24	13	<10	<10
	ASIR (95% CI)	0.0 (0.0–0.1)	0.0 (0.0–0.1)	0.0 (0.0–0.1)	0.1 (0.1–0.2)	0.0 (0.0–0.1)	0.0 (0.0–0.0)	0.0 (0.0–0.1)
Ependymal tumor	N	487	279	208	68	107	273	39
	ASIR (95% CI)	0.5 (0.5–0.5)	0.6 (0.5–0.6)	0.4 (0.4–0.5)	0.3 (0.2–0.4)	0.4 (0.3–0.4)	0.7 (0.7–0.8)	0.4 (0.3–0.5)
Neuronal and mixed neuronal-glial tumor	N	227	133	94	97	88	37	<10
	ASIR (95% CI)	0.2 (0.2–0.3)	0.3 (0.2–0.3)	0.2 (0.2–0.2)	0.4 (0.4–0.5)	0.3 (0.2–0.4)	0.1 (0.1–0.1)	0.0 (0.0–0.2)
Other glioma	N	297	160	137	50	77	133	37
	ASIR (95% CI)	0.3 (0.3–0.3)	0.3 (0.3–0.4)	0.3 (0.2–0.3)	0.2 (0.2–0.3)	0.3 (0.2–0.3)	0.4 (0.3–0.4)	0.3 (0.2–0.5)

Supplementary Table 5: Median overall survival (months) and 1-year and 5-year overall survival (%), by histopathology group, age group and period of diagnosis.
Survival not estimated if < 10 cases. Median survival not estimated if still > 50% of patients alive at end of follow-up. Follow-up time shortens in later periods (max 20 years for 2002-2006, max 15 years for 2007-2011, max 10 years for 2012-2016, max 5 years for 2017-2021).

Histopathology group	Period of diagnosis	Age at diagnosis	Cases	Median survival (months) (95% CI)	1-year overall survival (%) (95% CI)	5-year overall survival (%) (95% CI)
Glioblastoma	All years	All ages	3868	11 (11–12)	46.5 (45–48.1)	4.9 (4.2–5.6)
Glioblastoma	2002–2006	All ages	768	10 (9–10)	38.8 (35.3–42.2)	3.4 (2.3–4.9)
Glioblastoma	2007–2011	All ages	897	10 (10–11)	43 (39.8–46.2)	4 (2.9–5.4)
Glioblastoma	2012–2016	All ages	999	12 (11–12)	48.1 (45–51.2)	5.4 (4.1–7)
Glioblastoma	2017–2021	All ages	1204	13 (12–14)	52.8 (49.9–55.5)	6.2 (4.5–8.2)
Glioblastoma	All years	0–17 years	40	13 (8–19)	52.5 (36.1–66.5)	12.8 (4.5–25.7)
Glioblastoma	2002–2006	0–17 years	< 10			
Glioblastoma	2007–2011	0–17 years	12	7 (3–22)	33.3 (10.3–58.8)	8.3 (.5–31.1)
Glioblastoma	2012–2016	0–17 years	11	10 (4–31)	45.5 (16.7–70.7)	18.2 (2.9–44.2)
Glioblastoma	2017–2021	0–17 years	11	24 (7–47)	81.8 (44.7–95.1)	
Glioblastoma	All years	18–39 years	185	25 (19–33)	76.2 (69.3–81.7)	17.7 (12.3–23.9)
Glioblastoma	2002–2006	18–39 years	39	32 (17–42)	79.4 (63–89.1)	18.5 (8.2–32.2)
Glioblastoma	2007–2011	18–39 years	39	26 (15–38)	74.4 (57.6–85.3)	20.5 (9.6–34.2)
Glioblastoma	2012–2016	18–39 years	56	20 (14–33)	69.6 (55.8–79.9)	16.1 (7.9–26.8)
Glioblastoma	2017–2021	18–39 years	51	25 (16–36)	82.4 (68.8–90.4)	17.1 (6.5–31.9)
Glioblastoma	All years	40–69 years	2496	13 (13–13)	54 (52–55.9)	5.5 (4.6–6.6)
Glioblastoma	2002–2006	40–69 years	515	11 (10–12)	44.5 (40.1–48.7)	3.7 (2.3–5.6)
Glioblastoma	2007–2011	40–69 years	592	12 (11–13)	51.4 (47.3–55.3)	4.1 (2.7–5.9)
Glioblastoma	2012–2016	40–69 years	652	13 (13–14)	55.7 (51.8–59.4)	6.3 (4.6–8.4)
Glioblastoma	2017–2021	40–69 years	737	15 (14–16)	61.3 (57.6–64.7)	7.8 (5.5–10.7)
Glioblastoma	All years	70 years and over	1147	7 (6–7)	25.4 (22.9–27.9)	.9 (.5–1.8)
Glioblastoma	2002–2006	70 years and over	208	5 (4–6)	16.8 (12.1–22.2)	
Glioblastoma	2007–2011	70 years and over	254	6 (5–7)	19.3 (14.7–24.4)	1.2 (.3–3.2)
Glioblastoma	2012–2016	70 years and over	280	7 (6–8)	26.4 (21.4–31.7)	.7 (.1–2.4)
Glioblastoma	2017–2021	70 years and over	405	8 (7–9)	32.8 (28.3–37.4)	1.4 (.4–3.9)

Anaplastic astrocytoma	All years	All ages	510	24 (21–30)	73.3 (69.2–76.9)	34.5 (30.2–38.8)
Anaplastic astrocytoma	2002–2006	All ages	80	19 (14–30)	71.2 (60–79.9)	28.7 (19.3–38.9)
Anaplastic astrocytoma	2007–2011	All ages	99	18 (15–21)	64.6 (54.4–73.2)	23.2 (15.5–31.9)
Anaplastic astrocytoma	2012–2016	All ages	169	21 (19–26)	73.3 (65.9–79.3)	30 (23.3–37.1)
Anaplastic astrocytoma	2017–2021	All ages	162	62 (44–.)	79.6 (72.6–85.1)	50.8 (41.7–59.1)
Anaplastic astrocytoma	All years	0–17 years	11	16 (1–.)	81.8 (44.7–95.1)	27.3 (6.5–53.9)
Anaplastic astrocytoma	2002–2006	0–17 years	<10			
Anaplastic astrocytoma	2007–2011	0–17 years	<10			
Anaplastic astrocytoma	2012–2016	0–17 years	<10			
Anaplastic astrocytoma	2017–2021	0–17 years	<10			
Anaplastic astrocytoma	All years	18–39 years	168	94 (70–133)	95.2 (90.6–97.6)	65.9 (57.6–73)
Anaplastic astrocytoma	2002–2006	18–39 years	26	61 (20–133)	96.2 (75.7–99.4)	53.8 (33.3–70.6)
Anaplastic astrocytoma	2007–2011	18–39 years	31	64 (18–161)	83.9 (65.5–92.9)	51.6 (33–67.4)
Anaplastic astrocytoma	2012–2016	18–39 years	40	80 (47–.)	100 (.–.)	63.2 (45.9–76.3)
Anaplastic astrocytoma	2017–2021	18–39 years	71	>50% alive at end follow-up	97.2 (89.2–99.3)	78.7 (63.7–88)
Anaplastic astrocytoma	All years	40–69 years	268	19 (18–22)	69 (63.1–74.2)	22.7 (17.7–28.2)
Anaplastic astrocytoma	2002–2006	40–69 years	41	20 (13–25)	70.7 (54.3–82.2)	22 (10.9–35.5)
Anaplastic astrocytoma	2007–2011	40–69 years	49	17 (10–20)	63.3 (48.2–75)	12.2 (5–23)
Anaplastic astrocytoma	2012–2016	40–69 years	100	19 (17–23)	70 (60–78)	23 (15.3–31.6)
Anaplastic astrocytoma	2017–2021	40–69 years	78	24 (16–42)	70.5 (59–79.3)	31.5 (20.1–43.6)
Anaplastic astrocytoma	All years	70 years and over	63	6 (4–11)	31.7 (20.7–43.3)	3.2 (.6–9.8)
Anaplastic astrocytoma	2002–2006	70 years and over	10	3 (1–11)		
Anaplastic astrocytoma	2007–2011	70 years and over	15	4 (1–12)	26.7 (8.3–49.6)	
Anaplastic astrocytoma	2012–2016	70 years and over	26	7 (4–15)	46.2 (26.6–63.6)	7.7 (1.3–21.7)
Anaplastic astrocytoma	2017–2021	70 years and over	12	7 (2–16)	33.3 (10.3–58.8)	

Diffuse astrocytoma	All years	All ages	576	82 (68–97)	84.2 (81–86.9)	57.6 (53.3–61.7)
Diffuse astrocytoma	2002–2006	All ages	158	68 (48–105)	81 (74–86.3)	54.4 (46.3–61.8)
Diffuse astrocytoma	2007–2011	All ages	157	64 (44–89)	84.1 (77.4–88.9)	51 (42.9–58.5)
Diffuse astrocytoma	2012–2016	All ages	154	96 (78–.)	86.4 (79.9–90.9)	64.2 (56–71.2)
Diffuse astrocytoma	2017–2021	All ages	107	>50% alive at end follow-up	86 (77.8–91.3)	66.9 (53.6–77.2)
Diffuse astrocytoma	All years	0–17 years	47	>50% alive at end follow-up	93.6 (81.5–97.9)	77.5 (62.1–87.2)
Diffuse astrocytoma	2002–2006	0–17 years	19	>50% alive at end follow-up	94.7 (68.1–99.2)	78.9 (53.2–91.5)
Diffuse astrocytoma	2007–2011	0–17 years	16	>50% alive at end follow-up	87.5 (58.6–96.7)	62.5 (34.9–81.1)
Diffuse astrocytoma	2012–2016	0–17 years	<10			
Diffuse astrocytoma	2017–2021	0–17 years	<10			
Diffuse astrocytoma	All years	18–39 years	225	125 (101–158)	97.8 (94.7–99.1)	76.1 (69.6–81.5)
Diffuse astrocytoma	2002–2006	18–39 years	64	132 (88–174)	96.9 (88.1–99.2)	75 (62.5–83.9)
Diffuse astrocytoma	2007–2011	18–39 years	57	123 (64–168)	96.5 (86.7–99.1)	64.9 (51.1–75.7)
Diffuse astrocytoma	2012–2016	18–39 years	60	103 (90–.)	98.3 (88.8–99.8)	83 (70.7–90.5)
Diffuse astrocytoma	2017–2021	18–39 years	44	>50% alive at end follow-up	100 (.–.)	84.4 (49.3–96)
Diffuse astrocytoma	All years	40–69 years	253	50 (40–72)	80.2 (74.8–84.6)	47.5 (41.1–53.6)
Diffuse astrocytoma	2002–2006	40–69 years	65	28 (21–42)	70.8 (58.1–80.2)	35.4 (24–46.9)
Diffuse astrocytoma	2007–2011	40–69 years	71	50 (41–78)	84.5 (73.8–91.1)	46.5 (34.6–57.5)
Diffuse astrocytoma	2012–2016	40–69 years	76	79 (39–.)	84.2 (73.9–90.7)	56.6 (44.7–66.8)
Diffuse astrocytoma	2017–2021	40–69 years	41	>50% alive at end follow-up	80.5 (64.8–89.7)	58 (40.3–72.1)
Diffuse astrocytoma	All years	70 years and over	51	8 (6–11)	35.3 (22.6–48.2)	7.6 (1.8–19)
Diffuse astrocytoma	2002–2006	70 years and over	10	8 (0–11)	20 (3.1–47.5)	
Diffuse astrocytoma	2007–2011	70 years and over	13	8 (4–9)	23.1 (5.6–47.5)	
Diffuse astrocytoma	2012–2016	70 years and over	14	8 (2–25)	42.9 (17.7–66)	14.3 (2.3–36.6)
Diffuse astrocytoma	2017–2021	70 years and over	14	10 (3–30)	50 (22.9–72.2)	

Oligoastrocytic tumor	All years	All ages	249	109 (86–129)	89.2 (84.6–92.4)	64.2 (57.9–69.8)
Oligoastrocytic tumor	2002–2006	All ages	93	48 (29–90)	80.6 (71.1–87.3)	47.3 (36.9–57)
Oligoastrocytic tumor	2007–2011	All ages	78	94 (62–126)	93.6 (85.3–97.3)	65.4 (53.7–74.8)
Oligoastrocytic tumor	2012–2016	All ages	77	> 50% alive at end follow-up	94.8 (86.7–98)	83 (72.5–89.8)
Oligoastrocytic tumor	2017–2021	All ages	< 10			
Oligoastrocytic tumor	All years	0–17 years	12	> 50% alive at end follow-up	91.7 (53.9–98.8)	66.7 (33.7–86)
Oligoastrocytic tumor	2002–2006	0–17 years	< 10			
Oligoastrocytic tumor	2007–2011	0–17 years	< 10			
Oligoastrocytic tumor	2012–2016	0–17 years	< 10			
Oligoastrocytic tumor	All years	18–39 years	81	137 (109–186)	98.8 (91.6–99.8)	81.5 (71.2–88.4)
Oligoastrocytic tumor	2002–2006	18–39 years	36	93 (57–171)	97.2 (81.9–99.6)	66.7 (48.8–79.5)
Oligoastrocytic tumor	2007–2011	18–39 years	17	> 50% alive at end follow-up	100 (.–.)	94.1 (65–99.1)
Oligoastrocytic tumor	2012–2016	18–39 years	28	> 50% alive at end follow-up	100 (.–.)	92.9 (74.3–98.2)
Oligoastrocytic tumor	All years	40–69 years	137	92 (62–120)	87.6 (80.8–92.1)	59.6 (50.9–67.3)
Oligoastrocytic tumor	2002–2006	40–69 years	43	37 (19–113)	79.1 (63.6–88.5)	44.2 (29.2–58.2)
Oligoastrocytic tumor	2007–2011	40–69 years	52	66 (33–100)	92.3 (80.8–97)	57.7 (43.2–69.7)
Oligoastrocytic tumor	2012–2016	40–69 years	41	> 50% alive at end follow-up	90.2 (76.1–96.2)	77.8 (61.7–87.8)
Oligoastrocytic tumor	2017–2021	40–69 years	< 10			
Oligoastrocytic tumor	All years	70 years and over	19	15 (6–32)	57.9 (33.2–76.3)	21.1 (6.6–41)
Oligoastrocytic tumor	2002–2006	70 years and over	< 10			
Oligoastrocytic tumor	2007–2011	70 years and over	< 10			
Oligoastrocytic tumor	2012–2016	70 years and over	< 10			

Anaplastic oligodendroglioma	All years	All ages	145	130 (80–163)	90.3 (84.2–94.2)	67.7 (58.9–75)
Anaplastic oligodendroglioma	2002–2006	All ages	37	110 (18–159)	75.7 (58.5–86.5)	54.1 (36.9–68.4)
Anaplastic oligodendroglioma	2007–2011	All ages	31	65 (32–.)	83.9 (65.5–92.9)	54.8 (36–70.3)
Anaplastic oligodendroglioma	2012–2016	All ages	31	>50% alive at end follow-up	100 (.–.)	71 (51.6–83.7)
Anaplastic oligodendroglioma	2017–2021	All ages	46	>50% alive at end follow-up	100 (.–.)	91.7 (75.9–97.3)
Anaplastic oligodendroglioma	All years	0–17 years	<10			
Anaplastic oligodendroglioma	2002–2006	0–17 years	<10			
Anaplastic oligodendroglioma	All years	18–39 years	36	160 (76–.)	97.2 (81.9–99.6)	84.2 (65.8–93.2)
Anaplastic oligodendroglioma	2002–2006	18–39 years	<10			
Anaplastic oligodendroglioma	2007–2011	18–39 years	10	103 (3–.)	90 (47.3–98.5)	70 (32.9–89.2)
Anaplastic oligodendroglioma	2012–2016	18–39 years	<10			
Anaplastic oligodendroglioma	2017–2021	18–39 years	15	>50% alive at end follow-up	100 (.–.)	100 (.–.)
Anaplastic oligodendroglioma	All years	40–69 years	100	130 (75–164)	89 (81–93.8)	65.3 (54.5–74.2)
Anaplastic oligodendroglioma	2002–2006	40–69 years	27	52 (11–159)	70.4 (49.4–83.9)	48.1 (28.7–65.2)
Anaplastic oligodendroglioma	2007–2011	40–69 years	20	51 (15–.)	85 (60.4–94.9)	50 (27.1–69.2)
Anaplastic oligodendroglioma	2012–2016	40–69 years	26	>50% alive at end follow-up	100 (.–.)	73.1 (51.7–86.2)
Anaplastic oligodendroglioma	2017–2021	40–69 years	27	>50% alive at end follow-up	100 (.–.)	95.8 (73.9–99.4)
Anaplastic oligodendroglioma	All years	70 years and over	<10			
Anaplastic oligodendroglioma	2002–2006	70 years and over	<10			
Anaplastic oligodendroglioma	2007–2011	70 years and over	<10			
Anaplastic oligodendroglioma	2012–2016	70 years and over	<10			
Anaplastic oligodendroglioma	2017–2021	70 years and over	<10			

Oligodendroglioma	All years	All ages	312	> 50% alive at end follow-up	97.4 (94.9–98.7)	90.1 (85.9–93.1)
Oligodendroglioma	2002–2006	All ages	76	> 50% alive at end follow-up	96.1 (88.3–98.7)	88 (78.3–93.6)
Oligodendroglioma	2007–2011	All ages	82	> 50% alive at end follow-up	95.1 (87.5–98.1)	84.1 (74.3–90.5)
Oligodendroglioma	2012–2016	All ages	60	> 50% alive at end follow-up	100 (.–.)	93.3 (83.2–97.4)
Oligodendroglioma	2017–2021	All ages	94	> 50% alive at end follow-up	98.9 (92.7–99.8)	97.9 (91.7–99.5)
Oligodendroglioma	All years	0–17 years	< 10			
Oligodendroglioma	2002–2006	0–17 years	< 10			
Oligodendroglioma	2007–2011	0–17 years	< 10			
Oligodendroglioma	2017–2021	0–17 years	< 10			
Oligodendroglioma	All years	18–39 years	130	> 50% alive at end follow-up	98.5 (94–99.6)	95.7 (89.8–98.2)
Oligodendroglioma	2002–2006	18–39 years	35	> 50% alive at end follow-up	94.3 (79–98.5)	91.3 (75.5–97.1)
Oligodendroglioma	2007–2011	18–39 years	35	> 50% alive at end follow-up	100 (.–.)	97.1 (81.4–99.6)
Oligodendroglioma	2012–2016	18–39 years	18	> 50% alive at end follow-up	100 (.–.)	94.4 (66.6–99.2)
Oligodendroglioma	2017–2021	18–39 years	42	> 50% alive at end follow-up	100 (.–.)	100 (.–.)
Oligodendroglioma	All years	40–69 years	163	219 (178–.)	97.5 (93.6–99.1)	88.7 (82.3–92.8)
Oligodendroglioma	2002–2006	40–69 years	36	178 (103–.)	97.2 (81.9–99.6)	83.3 (66.6–92.1)
Oligodendroglioma	2007–2011	40–69 years	40	> 50% alive at end follow-up	92.5 (78.5–97.5)	80 (64–89.5)
Oligodendroglioma	2012–2016	40–69 years	39	> 50% alive at end follow-up	100 (.–.)	94.9 (81–98.7)
Oligodendroglioma	2017–2021	40–69 years	48	> 50% alive at end follow-up	100 (.–.)	97.9 (85.8–99.7)
Oligodendroglioma	All years	70 years and over	10	81 (11–.)	90 (47.3–98.5)	54.9 (18.7–80.6)
Oligodendroglioma	2002–2006	70 years and over	< 10			
Oligodendroglioma	2007–2011	70 years and over	< 10			
Oligodendroglioma	2012–2016	70 years and over	< 10			
Oligodendroglioma	2017–2021	70 years and over	< 10			

Pilocytic astrocytoma	All years	All ages	329	> 50% alive at end follow-up	96.7 (94–98.1)	92.9 (89.5–95.3)
Pilocytic astrocytoma	2002–2006	All ages	84	> 50% alive at end follow-up	96.4 (89.3–98.8)	94 (86.3–97.5)
Pilocytic astrocytoma	2007–2011	All ages	93	> 50% alive at end follow-up	96.8 (90.3–98.9)	91.4 (83.5–95.6)
Pilocytic astrocytoma	2012–2016	All ages	82	> 50% alive at end follow-up	97.6 (90.6–99.4)	93.9 (85.9–97.4)
Pilocytic astrocytoma	2017–2021	All ages	70	> 50% alive at end follow-up	95.7 (87.3–98.6)	89.7 (68.4–97)
Pilocytic astrocytoma	All years	0–17 years	213	> 50% alive at end follow-up	99.1 (96.3–99.8)	96.4 (92.6–98.3)
Pilocytic astrocytoma	2002–2006	0–17 years	54	> 50% alive at end follow-up	98.1 (87.6–99.7)	94.4 (83.8–98.2)
Pilocytic astrocytoma	2007–2011	0–17 years	58	> 50% alive at end follow-up	100 (.–.)	94.8 (84.8–98.3)
Pilocytic astrocytoma	2012–2016	0–17 years	47	> 50% alive at end follow-up	100 (.–.)	100 (.–.)
Pilocytic astrocytoma	2017–2021	0–17 years	54	> 50% alive at end follow-up	98.1 (87.6–99.7)	98.1 (87.6–99.7)
Pilocytic astrocytoma	All years	18–39 years	72	> 50% alive at end follow-up	94.4 (85.9–97.9)	91.5 (82.1–96.1)
Pilocytic astrocytoma	2002–2006	18–39 years	15	> 50% alive at end follow-up	93.3 (61.3–99)	93.3 (61.3–99)
Pilocytic astrocytoma	2007–2011	18–39 years	23	> 50% alive at end follow-up	95.7 (72.9–99.4)	91.3 (69.5–97.8)
Pilocytic astrocytoma	2012–2016	18–39 years	25	> 50% alive at end follow-up	96 (74.8–99.4)	92 (71.6–97.9)
Pilocytic astrocytoma	2017–2021	18–39 years	< 10			
Pilocytic astrocytoma	All years	40–69 years	41	215 (140–.)	90.2 (76.1–96.2)	82.4 (66.6–91.2)
Pilocytic astrocytoma	2002–2006	40–69 years	15	215 (96–.)	93.3 (61.3–99)	93.3 (61.3–99)
Pilocytic astrocytoma	2007–2011	40–69 years	11	> 50% alive at end follow-up	81.8 (44.7–95.1)	72.7 (37.1–90.3)
Pilocytic astrocytoma	2012–2016	40–69 years	< 10			
Pilocytic astrocytoma	2017–2021	40–69 years	< 10			
Pilocytic astrocytoma	All years	70 years and over	< 10			
Pilocytic astrocytoma	2007–2011	70 years and over	< 10			
Pilocytic astrocytoma	2012–2016	70 years and over	< 10			
Pilocytic astrocytoma	2017–2021	70 years and over	< 10			

Unique astrocytoma variant	All years	All ages	48	>50% alive at end follow-up	91.7 (79.3–96.8)	74.8 (59.9–84.9)
Unique astrocytoma variant	2002–2006	All ages	<10			
Unique astrocytoma variant	2007–2011	All ages	10	>50% alive at end follow-up	100 (.–.)	100 (.–.)
Unique astrocytoma variant	2012–2016	All ages	18	>50% alive at end follow-up	94.4 (66.6–99.2)	77.8 (51.1–91)
Unique astrocytoma variant	2017–2021	All ages	11	>50% alive at end follow-up	81.8 (44.7–95.1)	53 (20.9–77.3)
Unique astrocytoma variant	All years	0–17 years	24	>50% alive at end follow-up	100 (.–.)	91.7 (70.6–97.8)
Unique astrocytoma variant	2002–2006	0–17 years	<10			
Unique astrocytoma variant	2007–2011	0–17 years	<10			
Unique astrocytoma variant	2012–2016	0–17 years	10	>50% alive at end follow-up	100 (.–.)	90 (47.3–98.5)
Unique astrocytoma variant	2017–2021	0–17 years	<10			
Unique astrocytoma variant	All years	18–39 years	13	>50% alive at end follow-up	92.3 (56.6–98.9)	76.9 (44.2–91.9)
Unique astrocytoma variant	2002–2006	18–39 years	<10			
Unique astrocytoma variant	2007–2011	18–39 years	<10			
Unique astrocytoma variant	2012–2016	18–39 years	<10			
Unique astrocytoma variant	2017–2021	18–39 years	<10			
Unique astrocytoma variant	All years	40–69 years	<10			
Unique astrocytoma variant	2002–2006	40–69 years	<10			
Unique astrocytoma variant	2007–2011	40–69 years	<10			
Unique astrocytoma variant	2012–2016	40–69 years	<10			
Unique astrocytoma variant	2017–2021	40–69 years	<10			
Unique astrocytoma variant	All years	70 years and over	<10			
Unique astrocytoma variant	2002–2006	70 years and over	<10			
Unique astrocytoma variant	2012–2016	70 years and over	<10			
Unique astrocytoma variant	2017–2021	70 years and over	<10			

Ependymal tumor	All years	All ages	487	> 50% alive at end follow-up	97.3 (95.4–98.4)	91.1 (88–93.3)
Ependymal tumor	2002–2006	All ages	102	> 50% alive at end follow-up	99 (93.2–99.9)	91.2 (83.7–95.3)
Ependymal tumor	2007–2011	All ages	119	> 50% alive at end follow-up	99.2 (94.2–99.9)	90.7 (83.8–94.7)
Ependymal tumor	2012–2016	All ages	142	> 50% alive at end follow-up	95.1 (89.9–97.6)	90.1 (83.8–94)
Ependymal tumor	2017–2021	All ages	124	> 50% alive at end follow-up	96.8 (91.6–98.8)	91.1 (77.7–96.6)
Ependymal tumor	All years	0–17 years	68	> 50% alive at end follow-up	97.1 (88.7–99.3)	87.4 (76.3–93.6)
Ependymal tumor	2002–2006	0–17 years	13	> 50% alive at end follow-up	92.3 (56.6–98.9)	69.2 (37.3–87.2)
Ependymal tumor	2007–2011	0–17 years	15	> 50% alive at end follow-up	100 (.–.)	93.3 (61.3–99)
Ependymal tumor	2012–2016	0–17 years	20	> 50% alive at end follow-up	100 (.–.)	90 (65.6–97.4)
Ependymal tumor	2017–2021	0–17 years	20	> 50% alive at end follow-up	95 (69.5–99.3)	95 (69.5–99.3)
Ependymal tumor	All years	18–39 years	107	> 50% alive at end follow-up	100 (.–.)	95.9 (89.5–98.4)
Ependymal tumor	2002–2006	18–39 years	32	> 50% alive at end follow-up	100 (.–.)	96.9 (79.8–99.6)
Ependymal tumor	2007–2011	18–39 years	29	> 50% alive at end follow-up	100 (.–.)	96.4 (77.2–99.5)
Ependymal tumor	2012–2016	18–39 years	26	> 50% alive at end follow-up	100 (.–.)	96 (74.8–99.4)
Ependymal tumor	2017–2021	18–39 years	20	> 50% alive at end follow-up	100 (.–.)	94.7 (68.1–99.2)
Ependymal tumor	All years	40–69 years	273	> 50% alive at end follow-up	98.2 (95.7–99.2)	92.6 (88.6–95.2)
Ependymal tumor	2002–2006	40–69 years	51	> 50% alive at end follow-up	100 (.–.)	94.1 (82.9–98.1)
Ependymal tumor	2007–2011	40–69 years	68	> 50% alive at end follow-up	100 (.–.)	92.6 (83.2–96.9)
Ependymal tumor	2012–2016	40–69 years	81	> 50% alive at end follow-up	96.3 (89–98.8)	91.3 (82.6–95.8)
Ependymal tumor	2017–2021	40–69 years	73	> 50% alive at end follow-up	97.3 (89.5–99.3)	90.2 (69.9–97.1)
Ependymal tumor	All years	70 years and over	39	148 (93–191)	84.6 (68.9–92.8)	72.9 (55.1–84.5)
Ependymal tumor	2002–2006	70 years and over	< 10			
Ependymal tumor	2007–2011	70 years and over	< 10			
Ependymal tumor	2012–2016	70 years and over	15	> 50% alive at end follow-up	73.3 (43.6–89.1)	73.3 (43.6–89.1)
Ependymal tumor	2017–2021	70 years and over	11	> 50% alive at end follow-up	90.9 (50.8–98.7)	90.9 (50.8–98.7)

Neuronal and mixed neuronal-glial tumor	All years	All ages	227	>50% alive at end follow-up	96.9 (93.6–98.5)	93.3 (89–96)
Neuronal and mixed neuronal-glial tumor	2002–2006	All ages	48	>50% alive at end follow-up	97.9 (86.1–99.7)	95.8 (84.4–98.9)
Neuronal and mixed neuronal-glial tumor	2007–2011	All ages	41	>50% alive at end follow-up	97.6 (83.9–99.7)	92.6 (78.8–97.6)
Neuronal and mixed neuronal-glial tumor	2012–2016	All ages	62	>50% alive at end follow-up	96.8 (87.7–99.2)	91.9 (81.5–96.5)
Neuronal and mixed neuronal-glial tumor	2017–2021	All ages	76	>50% alive at end follow-up	96.1 (88.3–98.7)	93.9 (84.2–97.7)
Neuronal and mixed neuronal-glial tumor	All years	0–17 years	97	>50% alive at end follow-up	99 (92.9–99.9)	96.5 (89.3–98.9)
Neuronal and mixed neuronal-glial tumor	2002–2006	0–17 years	19	>50% alive at end follow-up	94.7 (68.1–99.2)	94.7 (68.1–99.2)
Neuronal and mixed neuronal-glial tumor	2007–2011	0–17 years	18	>50% alive at end follow-up	100 (.–.)	100 (.–.)
Neuronal and mixed neuronal-glial tumor	2012–2016	0–17 years	25	>50% alive at end follow-up	100 (.–.)	92 (71.6–97.9)
Neuronal and mixed neuronal-glial tumor	2017–2021	0–17 years	35	>50% alive at end follow-up	100 (.–.)	100 (.–.)
Neuronal and mixed neuronal-glial tumor	All years	18–39 years	88	>50% alive at end follow-up	98.9 (92.2–99.8)	97.4 (89.9–99.4)
Neuronal and mixed neuronal-glial tumor	2002–2006	18–39 years	18	>50% alive at end follow-up	100 (.–.)	100 (.–.)
Neuronal and mixed neuronal-glial tumor	2007–2011	18–39 years	18	>50% alive at end follow-up	94.4 (66.6–99.2)	88.5 (61.4–97)
Neuronal and mixed neuronal-glial tumor	2012–2016	18–39 years	26	>50% alive at end follow-up	100 (.–.)	100 (.–.)
Neuronal and mixed neuronal-glial tumor	2017–2021	18–39 years	26	>50% alive at end follow-up	100 (.–.)	100 (.–.)
Neuronal and mixed neuronal-glial tumor	All years	40–69 years	37	>50% alive at end follow-up	97.3 (82.3–99.6)	88.3 (71.7–95.5)
Neuronal and mixed neuronal-glial tumor	2002–2006	40–69 years	10	>50% alive at end follow-up	100 (.–.)	100 (.–.)
Neuronal and mixed neuronal-glial tumor	2007–2011	40–69 years	<10			
Neuronal and mixed neuronal-glial tumor	2012–2016	40–69 years	10	>50% alive at end follow-up	90 (47.3–98.5)	80 (40.9–94.6)
Neuronal and mixed neuronal-glial tumor	2017–2021	40–69 years	12	>50% alive at end follow-up	100 (.–.)	87.5 (38.7–98.1)
Neuronal and mixed neuronal-glial tumor	All years	70 years and over	<10			
Neuronal and mixed neuronal-glial tumor	2002–2006	70 years and over	<10			
Neuronal and mixed neuronal-glial tumor	2012–2016	70 years and over	<10			
Neuronal and mixed neuronal-glial tumor	2017–2021	70 years and over	<10			
Other glioma	All years	All ages	297	70 (43–113)	75.1 (69.8–79.6)	51.4 (45.4–57)
Other glioma	2002–2006	All ages	87	111 (48–171)	79.3 (69.2–86.4)	55 (44–64.8)
Other glioma	2007–2011	All ages	91	101 (48–.)	79.1 (69.2–86.1)	58.2 (47.4–67.6)
Other glioma	2012–2016	All ages	50	>50% alive at end follow-up	76 (61.6–85.6)	56 (41.2–68.4)
Other glioma	2017–2021	All ages	69	19 (12–35)	63.8 (51.3–73.9)	32.9 (20.5–45.9)
Other glioma	All years	0–17 years	50	>50% alive at end follow-up	76 (61.6–85.6)	62 (47.1–73.8)
Other glioma	2002–2006	0–17 years	<10			
Other glioma	2007–2011	0–17 years	14	>50% alive at end follow-up	100 (.–.)	100 (.–.)
Other glioma	2012–2016	0–17 years	<10			
Other glioma	2017–2021	0–17 years	21	12 (8–18)	47.6 (25.7–66.7)	23.8 (8.7–43.1)
Other glioma	All years	18–39 years	77	>50% alive at end follow-up	94.8 (86.7–98)	76.9 (65.4–85)
Other glioma	2002–2006	18–39 years	31	>50% alive at end follow-up	90.3 (72.9–96.8)	77.2 (58–88.4)
Other glioma	2007–2011	18–39 years	18	>50% alive at end follow-up	100 (.–.)	83.3 (56.8–94.3)
Other glioma	2012–2016	18–39 years	13	>50% alive at end follow-up	100 (.–.)	84.6 (51.2–95.9)
Other glioma	2017–2021	18–39 years	15	>50% alive at end follow-up	93.3 (61.3–99)	55 (17.3–81.5)
Other glioma	All years	40–69 years	133	40 (25–91)	78.2 (70.2–84.3)	43.9 (35.1–52.2)
Other glioma	2002–2006	40–69 years	40	48 (19–125)	82.5 (66.8–91.2)	42.5 (27.1–57)
Other glioma	2007–2011	40–69 years	50	32 (17–145)	76 (61.6–85.6)	44 (30.1–57.1)
Other glioma	2012–2016	40–69 years	24	41 (13–.)	75 (52.6–87.9)	50 (29.1–67.8)
Other glioma	2017–2021	40–69 years	19	26 (15–.)	78.9 (53.2–91.5)	
Other glioma	All years	70 years and over	37	4 (2–6)	21.6 (10.2–35.8)	8.6 (1.9–21.9)
Other glioma	2002–2006	70 years and over	<10			
Other glioma	2007–2011	70 years and over	<10			
Other glioma	2012–2016	70 years and over	<10			
Other glioma	2017–2021	70 years and over	14	6 (1–14)	35.7 (13–59.4)	
